# Infrared and Raman spectra of lignin substructures: Dibenzodioxocin

**DOI:** 10.1002/jrs.5808

**Published:** 2020-01-03

**Authors:** Peter Bock, Paula Nousiainen, Thomas Elder, Markus Blaukopf, Hassan Amer, Ronald Zirbs, Antje Potthast, Notburga Gierlinger

**Affiliations:** ^1^ Institute of Biophysics University of Natural Resources and Life Sciences Vienna Austria; ^2^ Department of Chemistry University of Helsinki Helsinki Finland; ^3^ USDA Forest Service Southern Research Station Auburn Alabama; ^4^ Institute of Organic Chemistry University of Natural Resources and Life Sciences Vienna Austria; ^5^ Institute of Chemistry of Renewable Resources University of Natural Resources and Life Sciences Vienna Austria; ^6^ Department of Natural and Microbial Products Chemistry National Research Centre Giza Egypt; ^7^ Institute of Biologically Inspired Materials University of Natural Resources and Life Sciences Vienna Austria

**Keywords:** lignin, dibenzodioxocin, Raman spectroscopy, FT‐IR, dehydrogenation, polymer

## Abstract

Vibrational spectroscopy is a very suitable tool for investigating the plant cell wall in situ with almost no sample preparation. The structural information of all different constituents is contained in a single spectrum. Interpretation therefore heavily relies on reference spectra and understanding of the vibrational behavior of the components under study. For the first time, we show infrared (IR) and Raman spectra of dibenzodioxocin (DBDO), an important lignin substructure. A detailed vibrational assignment of the molecule, based on quantum chemical computations, is given in the Supporting Information; the main results are found in the paper. Furthermore, we show IR and Raman spectra of synthetic guaiacyl lignin (dehydrogenation polymer—G‐DHP). Raman spectra of DBDO and G‐DHP both differ with respect to the excitation wavelength and therefore reveal different features of the substructure/polymer. This study confirms the idea previously put forward that Raman at 532 nm selectively probes end groups of lignin, whereas Raman at 785 nm and IR seem to represent the majority of lignin substructures.

## Graphical abstract

Lignin is a heterogeneous aromatic plant polymer and can be studied in situ in the plant cell wall using infrared (IR) and Raman microscopy. Analysis relies on the availability and understanding of reference spectra. IR and Raman spectra of dibenzodioxocin, a lignin substructure, are shown, leading to a new assignment of lignin. Results strengthen the idea that end groups can be highlighted using Raman at 532 nm, whereas the entire polymer can be studied using Raman at 785 nm and IR spectroscopy.

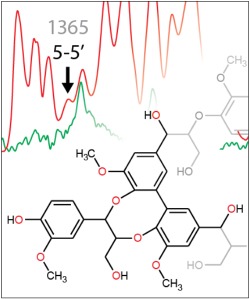

## 1 INTRODUCTION

Vibrational spectroscopy is a very suitable method for plant cell wall research, because chemical information can be related to spatial information and measurements can be performed on samples containing several cells. Especially Raman microscopy turned out to be very useful, because it is possible to analyze samples in the native state. With this technique, a laser is used to excite the sample and Raman scattering is recorded. Compared with other techniques, this method is virtually noninvasive and also requires little sample preparation. Different tissues and organelles can be studied simultaneously, with information gained on anatomical structure and chemical composition. This is done by rasterizing the sample and acquiring a spectrum on each pixel of the image.^1^, ^2^, ^3^ Infrared (IR) spectroscopy was long time limited by instrument resolution but, in combination with atomic force microscopy (AFM‐IR),^4^, ^5^, ^6^ is now able to obtain spectra at a 25 nm resolution.^7^ Cell corners can now be probed by both Raman and IR, which makes it possible to acquire lignin spectra in situ.

Lignin is the second most abundant plant polymer, and contents of 20–40% are usually found in wood.^8^ In the plant cell wall, it can be distinguished from other constituents by IR and Raman microscopy.^9^, ^10^, ^11^, ^12^ Published work has mainly focused on the estimation of ethylenic residues (cinnamyl alcohols and aldehydes)^13^, ^14^, ^15^, ^16^, ^17^, ^18^, ^19^ and determination of S/G ratios^20^, ^21^, ^22^, ^23^, ^24^, ^25^, ^26^, ^27^, ^28^ in lignin. However, these are only selected features of the lignin polymer; for example, cinnamaldehyde end groups only account for about 4% of lignin substructures.^29^, ^30^, ^31^, ^32^ To the best of our knowledge, in none of the studies using Raman or IR spectroscopy, abundant lignin linkages like ß‐O‐4 or ß‐ß were studied. This may be because studies often (have to) rely on band assignments available in the literature. Because no assignments exist for the latter linkages, they are also not studied. Another reason for this might be that conjugated aromatic structures show stronger scattering behavior and are therefore more dominant in the Raman spectrum, hence easier to identify.^33^, ^34^, ^35^, ^36^, ^37^ This makes Raman spectroscopy a very suitable tool for probing such lignin substructures.^38^ This could also explain that despite the number of lignin substructures known, only coniferyl alcohol and coniferyl aldehyde have had spectra assigned to date. Paired with our findings^38^ that mainly conjugated substructures are present in the Raman spectrum, this paper addresses biphenyl (5‐5′ coupled) substructures, which can benefit from these enhancing effects. Out of these, dibenzodioxocin (DBDO) is expected to give the strongest Raman signal, because the biphenyl rings should be sufficiently planar. Their amount is estimated by nuclear magnetic resonance (NMR) studies^29^, ^39^, ^40^ to be around 5%, and they were found across a variety of plant lignins, including those of softwoods, hardwoods, grasses, and legumes.^41^

Given that recently DBDO was considered as end points in relatively short polymer chains,^42^ it seems a good time to also deal with its vibrational spectra. In addition, here, we report spectra of a G‐based dehydrogenation polymer (G‐DHP), which should serve as a lignin model and reevaluate its previous literature assignments of the IR bands. This, together with our previous work, updates the lignin band assignments to the current research level.

## 2 METHODS

### 2.1 Synthesis of the model compound DBDO

**Figure 1:**
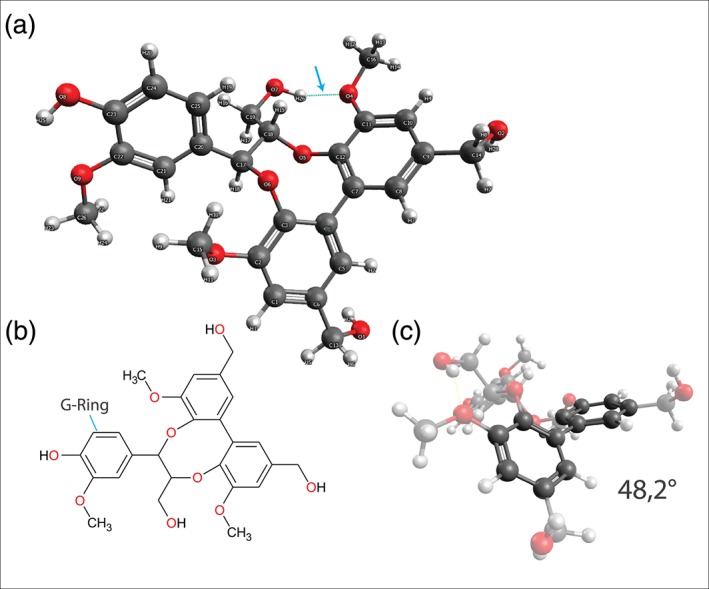
(a) Lowest energy conformer of DBDO after 1,000 steps of a Monte Carlo search performed with MMFF minimization. The arrow points to a hydrogen bond. (b) Chemical structure of DBDO. (c) Dihedral angle of the biphenyl unit [Colour figure can be viewed at http://wileyonlinelibrary.com]

2,2′‐((5,5′‐Bis (hydroxymethyl)‐3,3′‐dimethoxy‐[1,1′‐biphenyl]‐2,2′‐diyl)bis (oxy))bis(1‐(4‐hydroxy‐3‐methoxyphenyl)propane‐1,3‐diol (in the following only, DBDO; see Figure 1) was synthesized from coniferyl alcohol (0.9 g, 5.1 mmol) and dehydrodivanillyl alcohol (1.4 g, 4.6 mmol) by oxidative coupling according to Karhunen et al.,^43^ with 27% yield of the purified product achieved.

All reactions were followed by TLC. The model compound was isolated using standard extraction procedures, and the product was purified by high‐pressure silica flash column chromatography (Biotage SB4 instrument, Uppsala, Sweden) before use. The product was identified, and its purity was checked by NMR spectroscopy, using Varian Inova 500 spectrometer (Palo Alto, CA, USA); ^1^H (500 MHz) and ^13^C (125 MHz) in acetone‐d6. The purity was checked by HPLC‐MS using Agilent 1260 LC equipped with SQ‐MS and UV diode array detector. The separation was done using Zorbax SB‐C18 RRHT (2.1 × 50 mm, 1.8 μm) column and acetonitrile gradient with 0.1% formic acid. The M‐1 ions were detected for DBDO (483 *m*/*z*). Prior to Raman and IR measurements, the compound was placed under high vacuum (0.1 mbar) for 48 hr and then again run through LC‐MS (Shimadzu LC10, Shimadzu Austria, Korneuburg) equipped with a Shimadzu LCMS‐2020 mass detector and an Alltech ELSD3300 detector. Samples were analyzed over an Agilent XDB C18 (150 × 4.6 mm) column employing an acetonitrile/water gradient from 5% to 100% acetonitrile over 10 column volumes. Its identity was then cross‐checked by NMR. For this, samples were measured with a Bruker Topspin 3.5 pl6 software suite on a Bruker AVANCE III console with a 600‐MHz magnet. For structure verification, standard Bruker pulse programs for ^1^H, ^13^C, HSQC, HMBC, COSY, and TOCSY were utilized. Spectra were measured in MeOD and referenced internally to the solvent residual signal at either 3.31 ppm (^1^H) or 49.0 ppm (^13^C) and can be found in the Supporting Information.

### 2.2 Synthesis of the G‐DHP lignin

One gram of coniferyl alcohol synthesized according to Amer et al.^44^ dissolved in 10 ml of acetone was gently mixed with 100 ml of phosphate buffer (pH 6.6) and slowly (flow rate of 2 ml/hr) added with a syringe pump to the vigorously stirred solution of phosphate buffer (150 ml) containing horseradish peroxidase (7 mg, 220 units/mg) at 20°C. In parallel, the hydrogen peroxide solution (78 mmol, 2 ml of 30% *w*/*w* in 200 ml of deionized water) was added to the reaction mixture at a flow rate of 2.5 ml/hr. Then the entire mixture was purged by nitrogen and stirred for additional 20 hr. The reaction mixture was centrifuged at 5,000 rpm, and the precipitate was washed with deionized water (3×). It was then suspended in deionized water, freeze‐dried, and extracted with 100 ml of tetrahydrofuran to remove low molar mass residues.

### 2.3 Thermal gravimetric analysis

Thermal gravimetric analysis (TGA) of DBDO was performed on a Mettler Toledo TGA/DSC1 with 100‐ml min^−1^ nitrogen as purge gas and a heating rate of 10°C min^−1^ from 25°C to 250°C. The TGA results are shown in the Supporting Information.

### 2.4 Computational details

Given the number of rotatable bonds and ring flexibility of DBDO, a conformational search of the model compounds was performed using a 1,000‐step Monte Carlo search with MMFF minimization, as implemented in Spartan'16.^45^ The unique conformations identified were further refined with PM6 semiempirical optimization, also in Spartan'16. Density functional theory calculations were then performed on the 10 lowest energy conformation from the PM6 step using the B3LYP functional, the 6‐311G basis set, and the GD3 empirical dispersion correction, all within Gaussian 16, Revision A.03.^46^ Default values for optimization and grid size were used. The lowest energy conformation from the density functional theory calculations was used in our work.

Calculations of DBDO and a number of substructures (see Supporting Information) were also run with GAMESS^47^, ^48^ and performed on a work station running Microsoft Windows © 10, 64 bit. The version of the program was gamess.2016‐pgi‐linux‐mkl.exe. All calculations were done with the SCF‐DFT functional B3LYP with the 6‐311G basis set. For visualization, the wxMacMolPlt program was used.^49^

### 2.5 Raman and IR measurements

As described in Bock and Gierlinger,^38^ Raman spectra were acquired using a confocal Raman microscope (alpha300RA, WITec, Germany) with a 20× air objective (NA 0.4, Carl Zeiss, Germany). Less than 1 mg of each model compound was mounted on a standard microscopy glass slide for Raman experiments. For 532‐nm experiments, the sample was excited with a linear polarized Sapphire SF laser (532 nm, Coherent, USA). The scattering was detected with an optic multifiber (50 μm) directed to a spectrometer (UHDS 300, WITec, Germany) equipped with blazed gratings (600 and 1,800 g/mm^−1^, BLZ 500 nm) and a CCD camera (Andor DV401 BV, Belfast, Northern Ireland); 785‐nm experiments were conducted on the same instrument, using a linear polarized XTRA II laser (785 nm, Toptica Photonics, Germany). The scattering was detected with an optic multifiber (100 nm) directed to a spectrometer (UHTS 300, WITec, Germany) equipped with blazed gratings (600 and 1,200 g/mm^−1^, BLZ 750 nm) and a CCD camera (Andor DU401 DD, Belfast, Northern Ireland). The Raman scattering was collected with two laser polarizations (0°, 90°) as well as with polarizers. A detailed overview of the spectra recorded is given in the Supporting Information.

IR spectra were obtained with a FT‐IR ATR spectrometer (Vertex 70, Bruker, Billerica, USA) with 16/32 scans. The samples were directly mounted on the ATR unit and measured with the pressure stamp. Five measurements were averaged, cut, and baseline corrected using OPUS 7.5 software (Bruker, USA).

## 3 RESULTS AND DISCUSSION

DBDO is solid at 293K (20°C); it is transparent with a slight yellow hue. Its melting point could not be determined using thermogravimetric analysis (see Supporting Information). This points to a complex decomposition reaction.

### 3.1 Molecular structure

The lowest energy conformer of DBDO alongside its chemical structure is shown in Figure 1a. It is in agreement with X‐ray data of similar molecules.^50^, ^51^ One methoxy group is not planar with the ring to which it is attached; this seems to be due to steric hindrance with the G‐ring. However, this is in contradiction to the aforementioned X‐ray studies, where the aryl‐methoxy groups are always found to be in plane with the ring.

A single hydrogen bond is calculated between the aliphatic hydroxyl group and the neighboring oxygen of the methoxy group.

The calculated dihedral angle of the biphenyl unit in DBDO is 48.2°. This is only a slight deviation from unsubstituted biphenyl in solution (34°–44°),^52^, ^53^, ^54^ likely because the twisting of the rings is constrained by the eight‐membered ring formed by linkage with coniferyl alcohol.

### 3.2 Raman and IR spectra of DBDO

**Figure 2:**
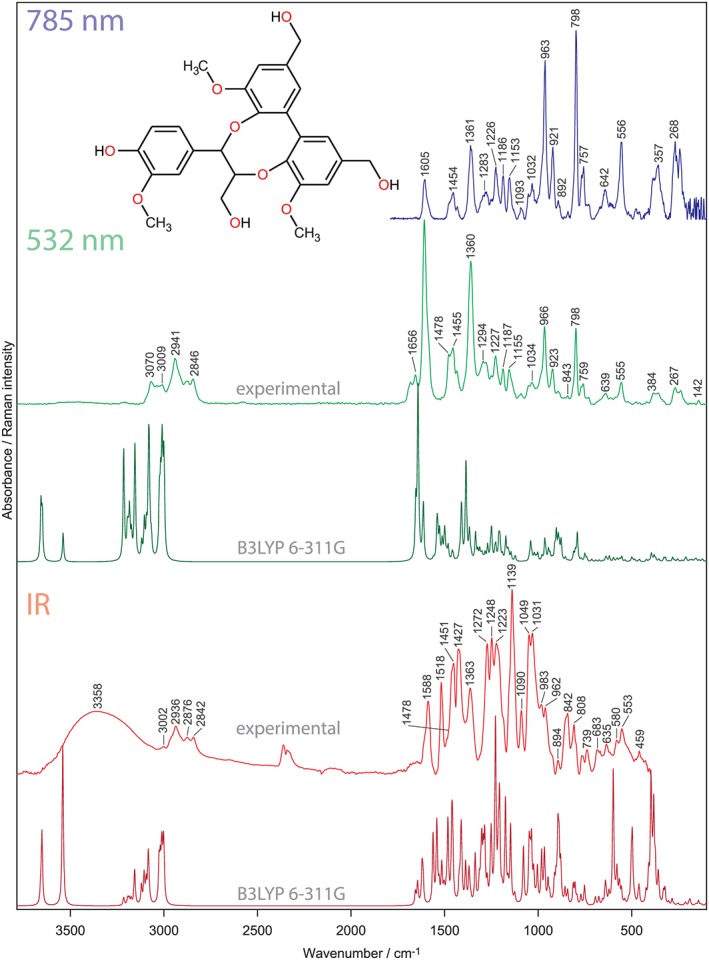
Infrared and Raman spectra of DBDO. The shape of Raman spectra differs between the excitation wavelengths. This relates to resonance enhancement, which is only created at 532 nm. Calculated, unscaled spectra are also shown [Colour figure can be viewed at http://wileyonlinelibrary.com]

Raman spectra of DBDO show only little fluorescence background, which is in agreement with previous studies where the rotation of individual rings about the coannular bond in 5‐5′ structures was deemed responsible for strong fluorescence observed in lignin spectra.^55^ Because this rotation is hindered in DBDO, the fluorescence is expected to be much smaller, as also observed (Figure 2).

A detailed discussion of the IR and Raman spectra is given in the Supporting Information, and only the most important bands are considered below. The Raman at 532 nm is mainly used in the text and referred to as “Raman” only; if the discussion extends to the spectra at 785 nm, this will be denoted.

The assignment is mainly based on the quantum chemical calculations of DBDO and several of its substructures. Ring modes are designated “Φ” and explained in more detail in the Supporting Information.

The C–H stretching region (3,100–2,700 cm^−1^) shows mainly bands of the aliphatic hydrogens, the strongest being the symmetric CH_2_ stretch of the methoxy group. The ring C–Hs (3,080–3,010 cm^−1^) are more clearly visible in the Raman spectrum, because in IR, the broad O–H stretch extends in this region.

DBDO also shows a typical Raman marker band for conjugated aromatic structures; this is the ring stretch at 1,608 cm^−1^, which is the strongest band in the spectrum. It can be inferred from the Raman at 532‐nm spectrum that the two biphenyl rings are still in conjugation with each other to such an extent and that the excitation frequency approaches an electronic transition. This is seen by the fact that this ring stretch is much stronger than the ring stretch at 798 cm^−1^. At 785 nm, the situation is reversed, and this enhancement is not seen anymore and the spectral shape is similar to that of unconjugated aromatic structures. Therefore, we attribute this solely to resonance enhancement. The contribution of the so‐called conjugation effect, which would be wavelength independent, is therefore negligible. This means that the polarizability is confined to the local coordinate of the ring mode. Our approach is in agreement with previous measurements of Raman intensities of conjugated systems.^34^

In IR, the band at around 1,500 cm^−1^ is normally referred to as marker band for phenyl rings. In DBDO, it shows only the G‐ring, as both combinations of the BP are downshifted into the region normally occupied by C–H bendings. The in‐phase combination is seen at 1,478 cm^−1^, next to C–H bending modes at 1,451 cm^−1^. This means that this substructure is not represented by the 1,507‐cm^−1^ lignin band!

The in‐phase C–X stretch of the BP is seen as strong band at 1,360 cm^−1^ in Raman. It is the only band that can be recognized also in lignin Raman spectra as being DBDO specific (see below). In the IR, typical G‐bands are recognized; these are 1,272 and 1,232 cm^−1^. C–H bendings are responsible for the strongest IR band at 1,139 cm^−1^. Two of the G‐ring markers fall together with two BP modes to cause this strong band. Various C–O stretchings are responsible for the bands ranging from 1,090 to 900 cm^−1^, which form a broad band complex in the IR spectrum. Interestingly, no typical band for the eight‐membered ring could be identified—this means that it is not possible to identify this structural feature of lignin also from the IR spectrum, although some contribution of its modes will be found in the range of 1,050 to 800 cm^−1^. The out‐of‐phase C–O stretch of the ring methoxy groups at 1,048 cm^−1^ has some contribution from this linkage, but because methoxy group vibrations are an inherent part of lignin spectra (except H‐ and C‐Lignin, which are built of p‐hydroxyphenyl and caffeyl alcohol units, respectively), this is of no specific diagnostic value.

Ring modes interacting with these stretches can be seen more readily in the Raman spectrum: 966 and 923 cm^−1^. The former band reaches similar intensity as the ring stretch typically seen for G‐ and S‐units at 798 cm^−1^. On the other hand, the C–H out‐of‐plane modes are only seen in the IR spectrum (900–800 cm^−1^). Below 700 cm^−1^, bands are seen to ride on a broad band caused by the O–H torsion modes. In Raman, there are less bands observed; they mainly stem from ring modes (639, 555, and 267 cm^−1^). The methoxy bending is also clearly visible at 384 cm^−1^.

### 3.3 Relevance for IR and Raman spectroscopy of cell walls

Up to now, mostly monomers^38^, ^56^, ^57^ and dimers^58^ have been used to explain the vibrational spectra of lignin. Although monomers have the advantage that the pure ring modes can be studied, some of the lignin linkages require a second or third unit—this is the case for DBDO. It is a next step in the quest for explaining the lignin polymer spectrum.

**Figure 3:**
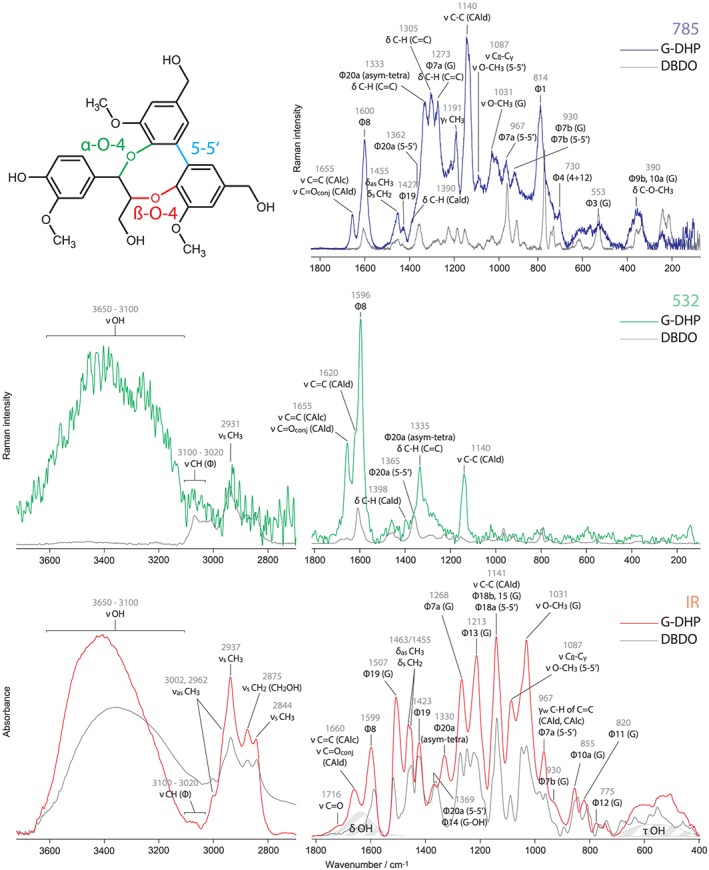
Raman and IR spectra of G‐DHP (dehydrogenation polymer) and DBDO. The region from 3,700 to 2,700 cm^−1^ has been scaled up to aid visibility. Assignments are given for the DHP. In the chemical structure of DBDO, different types of linkages are highlighted. The shaded area informs on how OH modes add to the spectrum; the shape was derived from the IR spectrum of water. G, G‐ring [Colour figure can be viewed at http://wileyonlinelibrary.com]

DBDO is therefore a well‐suited reference structure, especially for G‐lignin, because it already incorporates important function groups of lignin. These are a G‐ring, terminal CH_2_OH groups, and methoxy groups. It also includes a 5‐5′, α‐O‐4, and ß‐O‐4 linkage (see Figure 3); these alone account for about 80% of the total linkages in lignin as estimated by NMR^29^. It is therefore not surprising that the IR spectra of DBDO and G‐DHP match quite well. It is also helpful that G‐DHPs have an elevated number of cinnamyl alcohol end groups, which in turn can oxidize to aldehydes^41^—both end groups are well characterized in the Raman and IR spectra of wood.^59^, ^60^, ^61^, ^62^ Therefore, the assignment of the DHP spectrum is facilitated.

In Figure 3, vibrational spectra of DBDO are compared with those of G‐DHP. There is an interesting difference visible between the two excitation wavelengths of the Raman spectra. Whereas the Raman at 532‐nm spectrum of DBDO does not fit so well for reasons explained above, the Raman at 785 nm looks much more similar to the IR spectrum. However, the IR spectrum of DBDO fits very well to the IR spectrum of DHP, whereas the Raman at 785 nm fits is less so. Considering all three spectra, it is apparent that although they stem from the same substance, they show different things. As we have already noted in a previous publication,^38^ conjugated structures like coniferyl alcohol and aldehyde end groups are highlighted in the Raman at 532‐nm spectrum. On the contrary, Raman at 785 nm looks more similar to the IR spectrum, which means that both represent more the overall lignin polymer. C–H bendings of the methyl and methylene groups (~1,460 cm^−1^) are stronger in the IR spectrum; their rocking motions (1,191 cm^−1^) are only visible in the Raman at 785‐nm spectrum. It also shows the C–H bendings of ethylenic residues (~1,340–1,300 cm^−1^), although the ring modes of the prevalent G‐rings (1,268, 1,213 cm^−1^) are not really seen in contrast to the IR spectrum, where they are marker bands.

For lignin analysis, this means that it is worthwhile to have all these spectra at hand, because information of lignin substructures is spread over the different effects and needs to be collected to avoid misinterpretation. This is also the case for DBDO, which is visible in Raman at 532 nm and can be identified by the band at ~1,360 cm^−1^. In Raman at 785 nm, the band at 967 cm^−1^ is indicative for this substructure; however, it has also contribution from other 5‐5′ structures (not shown). In IR, the band at 1,369 cm^−1^ has also contribution from G‐rings (4‐OH, i.e., end groups also) and the 967 cm^−1^ should be mainly seen as being indicative for ethylenic residues. The IR band at 1,507 cm^−1^ is indicative of G‐structures due to the downshift of Φ19 of biphenyl structures.

In our last paper we supplied an updated assignment of the Raman spectrum of lignin.^38^ In this paper, based on our findings for DBDO, we present an updated table of the IR spectrum (see Table 1).

**Table 1:** Overview of the IR band assignments of G‐lignin. Note that Hergert studied milled wood lignins and Faix studied Western hemlock native lignin. Both authors gave assignments for lignin in general, whereas our assignment work is based on G‐DHP. Φ = Ring mode in Wilson/Varsanyi notation. Two charts depicting all ring modes for asym‐trisubstituted and asym‐tetrasubstituted rings can be found in the Supporting Information

Wavenumber (G‐DHP)	Hergert^64^	Faix^65^	This work
~3,400	O–H stretching (H‐bonded)	O–H stretch	O–H stretch (H‐bonded)
3,086			C–H stretch of aromatic ring
3,065			C–H stretch of aromatic ring Φ2
3,035			C–H stretch of aromatic ring
3,002		C–H stretch in methyl and methylene groups	C–H stretch of OCH_3_
2,960		C–H stretch in methyl and methylene groups	Asymmetric C–H stretch of OCH_3_
2,937	C–H stretching (methoxyl groups and side‐chain CH) (Assigned to 2,920 in original publication)	C–H stretch in methyl and methylene groups	Symmetric C–H stretch of OCH_3_ Antisymmetric stretch of CH_2_OH
2,875	C–H stretching (methoxyl groups and side‐chain CH)	C–H stretch in methyl and methylene groups	Symmetric C–H stretch of CH_2_OH
2,844		C–H stretch in methyl and methylene groups	Symmetric C–H stretch of OCH_3_
1,720	C=O stretching of aliphatic ketone	C=O stretch in unconjugated ketone, carbonyl, and in ester groups (frequently of carbohydrate origin); conjugated aldehydes and carboxylic acids absorb around and below 1,700 cm^−1^	C=O stretch of unconjugated carbonyls
1,660	C=O stretching of p‐substituted aryl ketone	C=O stretch; in conjugated p‐subst. aryl ketones; strong electronegative substituents lower the wavenumber	C=O stretch of conjugated carbonyls (i.e., coniferyl aldehyde, carbonyls in α‐position, carbonyls of quinone methide) C=C stretching of coniferyl alcohol
1,599	C=C skeletal vibrations (aromatic ring)	Aromatic skeletal vibrations plus C=O stretch; S > G; G condensed > G etherified	Ring stretch Φ8b of G‐rings and Φ8a of S‐rings^a^
1,507	C=C skeletal vibrations (aromatic ring)	Aromatic skeletal vibrations; G > S	Ring stretch Φ19b of G‐rings and Φ19a of S‐rings^a^
1,463	C–H deformation (asymmetric)	C–H deformations; asym. In –CH_3_ and –CH_2_–	C–H bending of OCH_3_ and CH_2_
1,455		C–H deformations; asym. In –CH_3_ and –CH_2_–	C–H bending of OCH_3_ and CH_2_
1,423		Aromatic skeletal vibrations combined with C–H in‐plane deform.	Ring stretch Φ19a of G‐rings and Φ19b of S‐rings^a^
1,369	C–H deformation (symmetric)	Aliphatic C–H stretch in CH_3_; not in OMe; phen. OH	Ring stretch Φ14 of 4‐OH‐G‐rings and S‐rings^a^ Ring stretch Φ20a of 5‐5′ structures
1,330		S‐ring plus G‐ring condensed (i.e., G‐ring substituted in pos. 5)	Ring stretch Φ20a of asymmetric‐tetrasubstituted rings (C–X; X=C, O)
1,268	C–O stretching aromatic (methoxyl)	G‐ring plus C=O stretch; G condensed > G etherified	Ring bend Φ7a of G‐rings
1,213	C–O stretching aromatic (phenol)	C–C plus C–O plus C=O stretch; G condensed > G etherified (authors give range from 1,221 to 1,230)	Ring bend Φ13 of G‐rings
1,190	Unassigned (methoxyl group)		C–H rocking of methoxy groups
1,141		Aromatic C–H in‐plane deformation; typical for G‐units; whereby G condensed > etherified (typical for S‐units): plus secondary alcohols plus C=O stretch	C–C stretch of coniferyl aldehyde C–H bend Φ18b, Φ15 of G‐rings CH bend Φ18a of 5‐5′ structures
1,087	C–O deformation (aliphatic ether or secondary hydroxyl)	C–O deformation in secondary alcohols and aliphatic ethers	C–C stretch of C_ß_ and C_γ_ C–O stretch of methoxy groups of 5‐5′ structures
1,031	C–O deformation (methoxyl group)	Aromatic C–H in‐plane deformation, G > S; plus C–O deform. In primary alcohols; plus C=O stretch (unconj.)	C–O stretch of methoxy groups of G‐rings
967	=CH out‐of‐plane deformation (trans)	–HC=CH– out‐of‐plane deform (trans)	C–H wagging of C=C of coniferyl aldehyde and alcohol In‐phase ring bend Φ7a of 5‐5′ structures
930	Unassigned (possibly OH out‐of‐plane deformation)	C–H out‐of‐plane; aromatic (authors give range from 915 to 925)	Ring bend Φ7b of G‐rings
855	C–H out‐of‐plane deformation (one H, aromatic ring)	C–H out‐of‐plane in positions 2, 5, and 6 of G‐units	C–H out‐of‐plane bend Φ10a of G‐rings
820	C–H out‐of‐plane deformation (two H, aromatic ring)	C–H out‐of‐plane in positions 2, 5, and 6 of G‐units	C–H out‐of‐plane bend Φ11 of G‐rings
775			Ring bend Φ12 of G‐rings

a Includes G‐units with substituents on ring position 5.

## 4 CONCLUSION

IR and Raman spectra of the lignin substructure DBDO were measured and assigned with the help of quantum chemical simulations. A synthetic guaiacyl lignin (G‐DHP) was also measured and compared with DBDO.

Raman spectra of DBDO and G‐DHP both differ with respect to the excitation wavelength and therefore reveal different features of the compound/polymer.

From this and from our previous work,^38^, ^63^ it can be deduced that Raman at 532 nm selectively probes lignin end groups like cinnamaldehydes, cinnamyl alcohols, and DBDOs. Raman at 785 nm and IR spectra represent the lignin units in the lignin polymer but show slightly differences, which may be further refined to give more diagnostic evidence of the different substructures in the future.

The mantra that IR should always be used in conjunction with Raman for detailed chemical analysis is repeated here with the addition that it is worthwhile to acquire Raman spectra at preresonant and nonresonant regimes for an additional insight into lignin.

## 5 FUNDING INFORMATION

Horizon 2020 Framework Programm, ERC Consolidator grant

Grant number: 681885

START grant of the Austrian Science Fund (FWF)

Grant number: Y‐728‐B16

## Supporting information

Data S1. A detailed characterization of DBDO can be found in the supplementary material. It includes interpretation of its IR, Raman and NMR spectra.
