# Supplementary material for: Infrared and Raman spectra of lignin substructures: Dibenzodioxocin
Source: J Raman Spectrosc. 2020 Jan 3;51(3):422–31. doi: 10.1002/jrs.5808 (PMC7079546; doi:10.1002/jrs.5808)
Supplement: Supplementary file 1 — Data S1. A detailed characterization of DBDO can be found in the supplementary material. It includes interpretation of its IR, Raman and NMR spectra. [file JRS-51-422-s001.pdf]

Supplementary material

Infrared and Raman spectra of lignin substructures: Dibenzodioxocin

Peter Bock[1], Paula Nousiainen[2], Thomas Elder[3], Markus Blaukopf[4], Hassan Amer[5,6], Ronald Zirbs[7], Antje Potthast[5] and Notburga Gierlinger[1]

[1]Institute of Biophysics, University of Natural Resources and Life Sciences, Vienna, Austria

[2]Laboratory of Organic Chemistry, University of Helsinki, Helsinki, Finland

[3]USDA Forest Service, Southern Research Station, Auburn, USA

[4]Institute of Organic Chemistry, University of Natural Resources and Life Sciences, Vienna, Austria

[5]Institute of Chemistry of Renewable Resources, University of Natural Resources and Life Sciences, Vienna, Austria

[6]Department of Natural and Microbial Products Chemistry, National Research Centre, Giza, Egypt.

[7]Institute of Biologically inspired materials, University of Natural Resources and Life Sciences, Vienna, Austria

Table of contents

Thermal gravimetric analysis (TGA) ... 3
Liquid chromatography - mass spectrometry (LC-MS) ... 4
Vibrational spectra of dibenzodioxocin ... 5
Molecular structures used for the assignment ... 5
Infrared and Raman spectra of dibenzodioxocin ... 18
Introductory notes on the vibrational analysis of biphenyls ... 22
Commented assignment of dibenzodioxocin ... 25
Nuclear magnetic resonance (NMR) spectra of dibenzodioxocin ... 42
UV-Vis spectrum of DBDO ... 45
Vibrational modes of substituted benzene rings ... 46
Literature ... 48

Thermal gravimetric analysis (TGA)

TGA showed no clear melting point, this points to a degradation reaction under elevated temperatures. The Graph is shown in Fig.1.

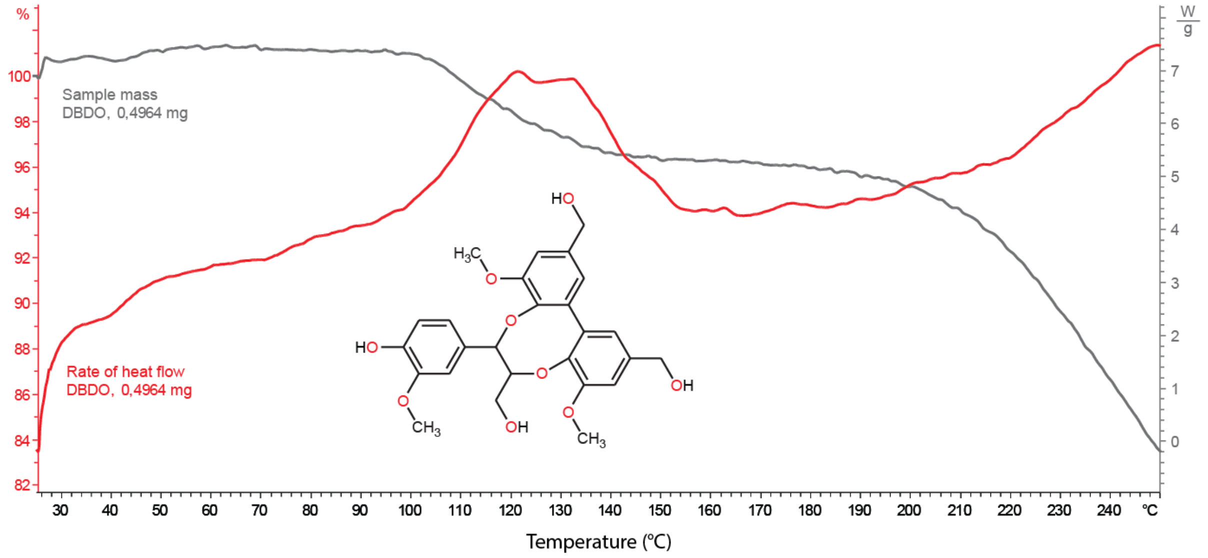

Figure 1 - TGA graph of DBDO.

Liquid chromatography - mass spectrometry (LC-MS)

Fig. 2 shows the LC-MS data.

ELSD trace

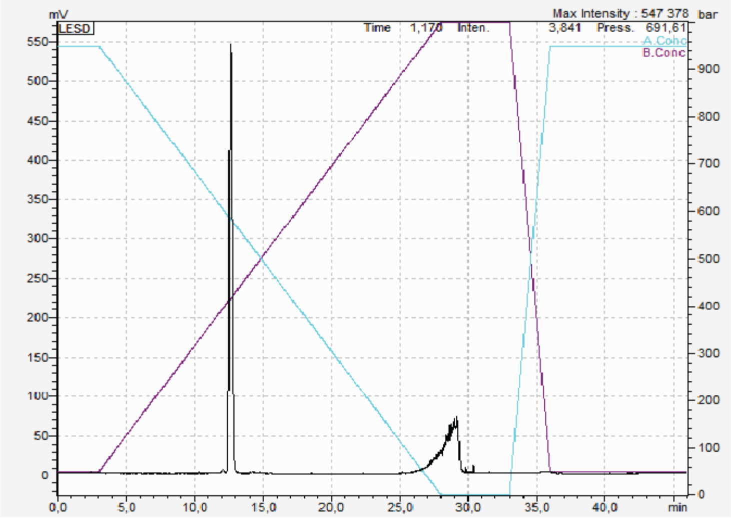

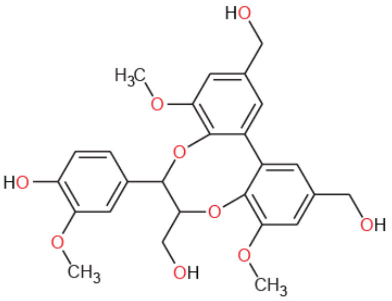

Exact mass: 484,17 g/mol

m/z trace (negative mode): 11.7 - 12.6 minutes

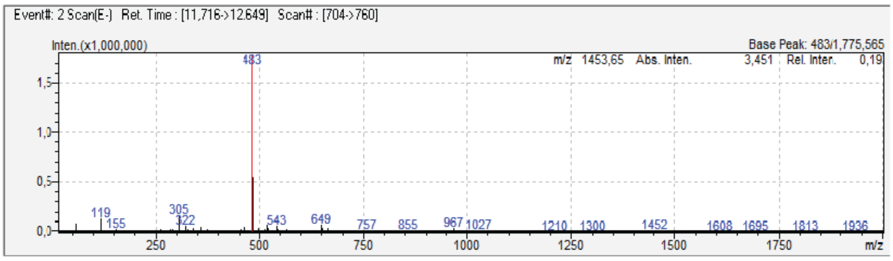

Figure 2 - LC-MS data of DBDO. Signal of the Evaporative Light Scattering Detector (ELSD) and mass to charge ratio.

Vibrational spectra of dibenzodioxocin

Molecular structures used for the assignment

Assigning vibrational spectra of a molecule requires not only a number of spectra of the molecule in questions as well as from similar compounds, it is also beneficial to have computations of similar structures at hand. This is, because, the heart of spectral interpretation is to assign a vibrational mode, which is a certain displacement pattern, to a band in the spectrum. However, it can happen that the calculated displacements are not understandable in terms that they cannot be described in established patterns, like those existing for the benzene ring. Coupling of modes for example can lead to a resulting displacement in which the contributing modes cannot be identified unequivocally. Changing the conformation or ring substituents can enhance interpretation, as in this cases calculated modes might be more characteristic. Furthermore this might give an idea on how a mode reacts on changing geometry or chemical environment, both of which make the overall interpretation more robust.

Some structures were calculated with GAMESS, others with GAUSSIAN ©, and some with both.

Calculations with GAMESS[1,2] were performed on a work station running Microsoft Windows © 10, 64 bit. The version of the program was: gamess.2016-pgi-linux-mkl.exe. All calculations were done with the SCF-DFT functional B3LYP with the 6-311G basis set. For visualization, the wxMacMolPlt program was used.[3] Optimized structures were used for calculation of the hessian matrix.

For calculations with GAUSSIAN[4], first a conformational search of the model compounds was performed using a 1000 step Monte Carlo search with MMFF minimization, as implemented in Spartan'16[5]. The unique conformations identified were further refined with PM6 semi-empirical optimization, also in Spartan'16. Density functional theory calculations were then performed on the 10 lowest energy conformation from the PM6 step using the B3LYP functional, the 6-311G basis set and the GD3 empirical dispersion correction, all within Gaussian 16, Revision A.03. Default values for optimization and grid size were used. The lowest energy conformation from the density functional theory calculations was used in the current work.

The structures which were used for the interpretation of the biphenyls, are listed and shown below. The G-ring was much less of an issue, because we have clearer understanding of this substructure

Table 1 – Names of the model structures, the software used to calculate the vibrational spectrum and the abbreviation for this compound used in the assignment text.

Name	Software used	Abbreviation used in text
5-5' basic structure	Gamess	5-5BP
DBDO SS	Gamess	Gms-DBDO
DBDO SS	Gaussian	Gau-DBDO-trans
DBDO RS	Gaussian	Gau-DBDO-cis
DBDO backbone	Gamess	Gms-BP-unit
DBDO backbone	Gaussian	Gau-BP-unit
Biphenyl unit 0°	Gamess	BP0
Biphenyl unit 45°	Gamess	BP45
Biphenyl unit 90°	Gamess	BP90
G-Ring	Gamess	
G-Ring	Gaussian	
2-Methoxy-4-propylphenol	Gamess	

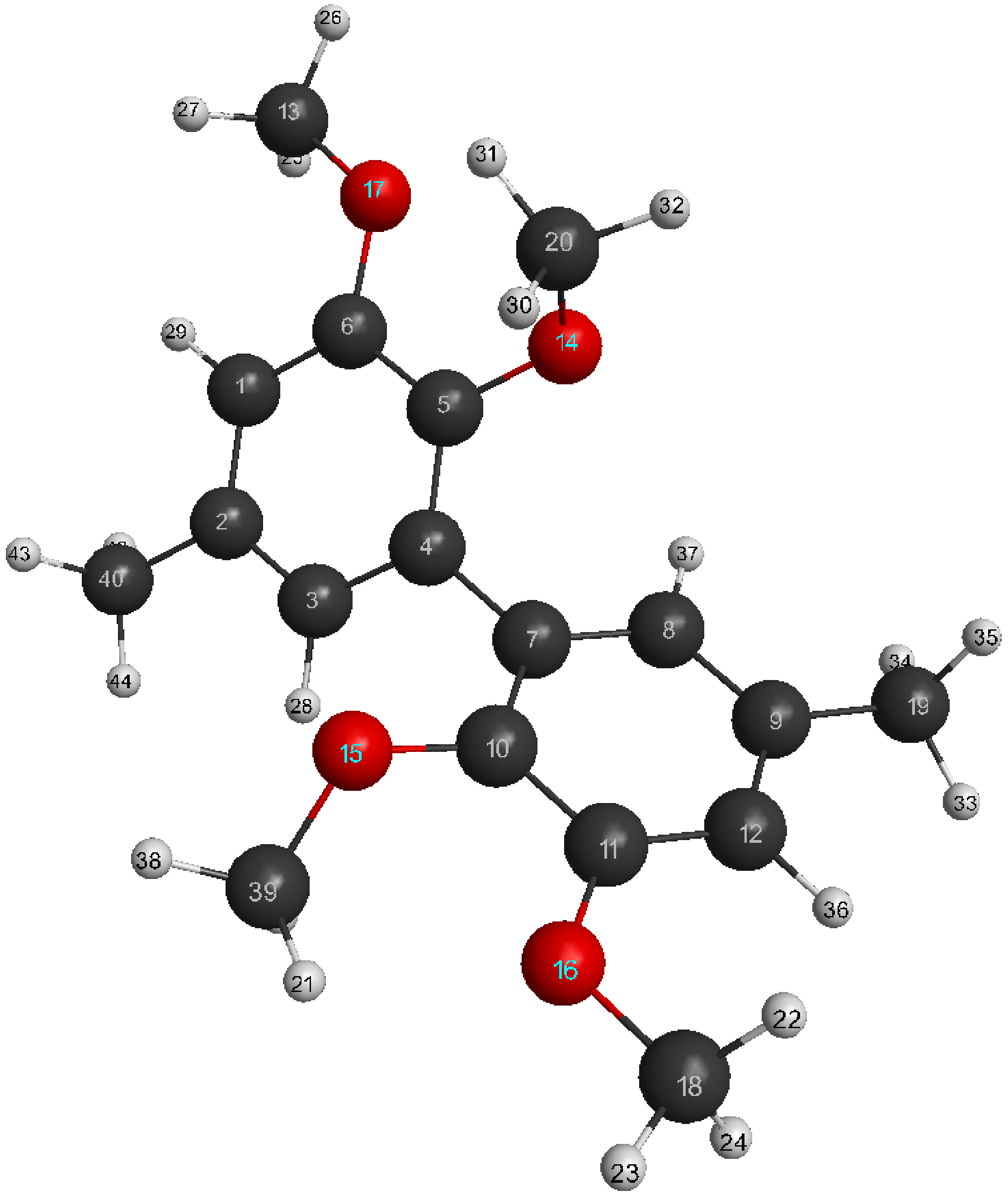

Figure 3 – Molecular model of the 5-5’ basis structure.

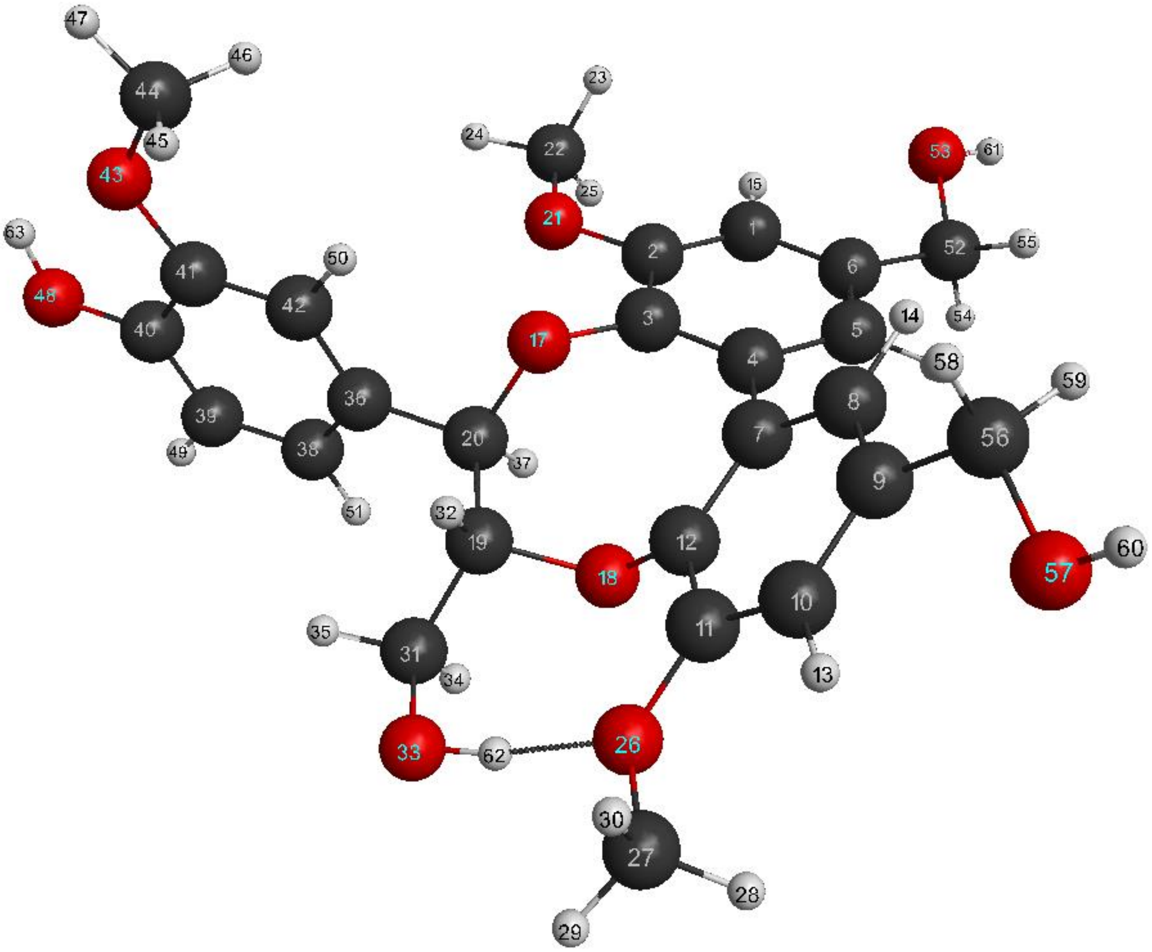

*Figure 4 – Molecular structure of DBDO SS (Dibenzodioxocin, SS-configuration) calculated with GAMESS. This configuration, also called **trans**, refers to the orientation of hydrogens no. 32 and 37.*

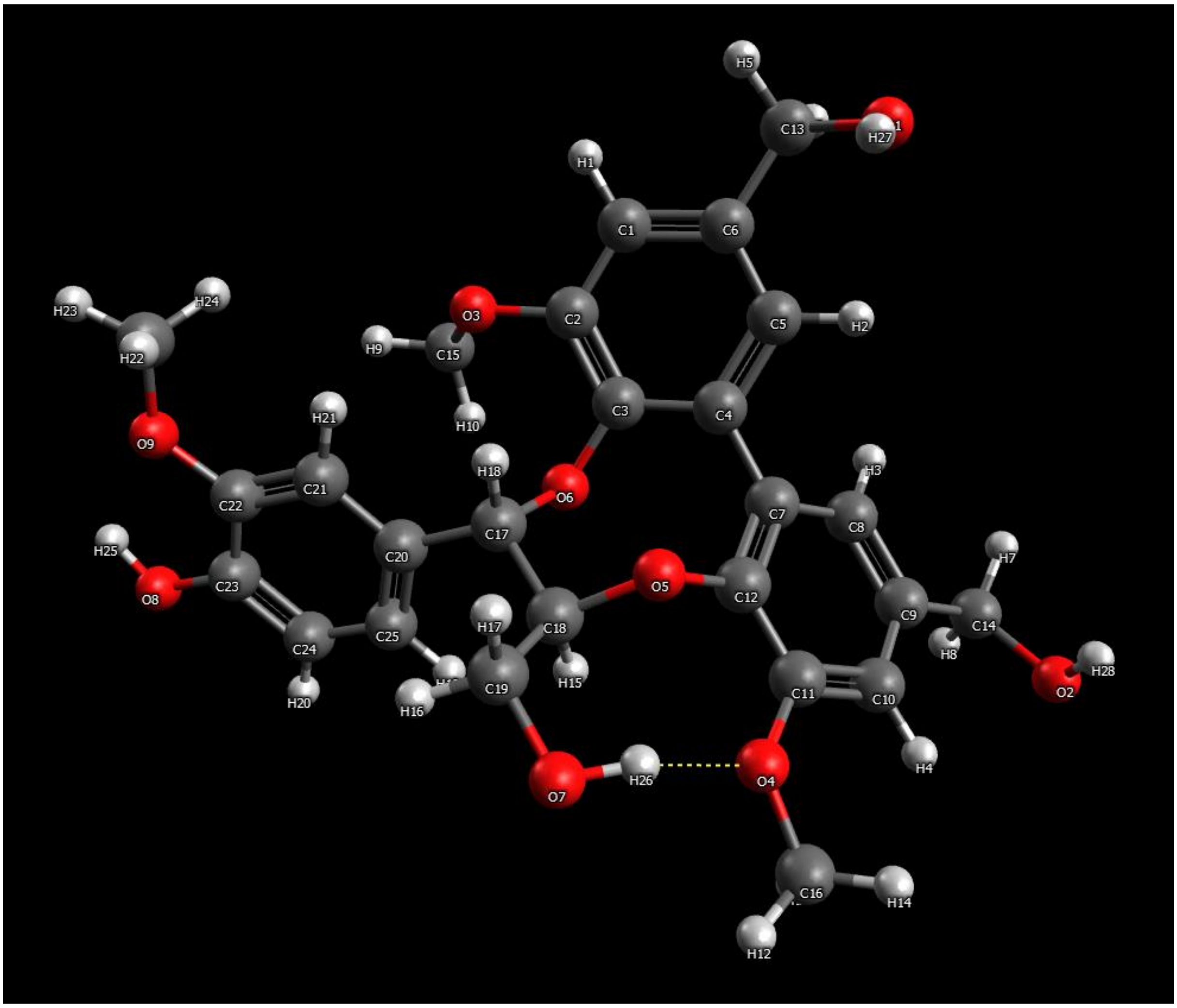

Figure 5 - Calculated structure of DBDO SS determined with Spartan'16 and Gaussian. This molecule is also in SS (trans) configuration.

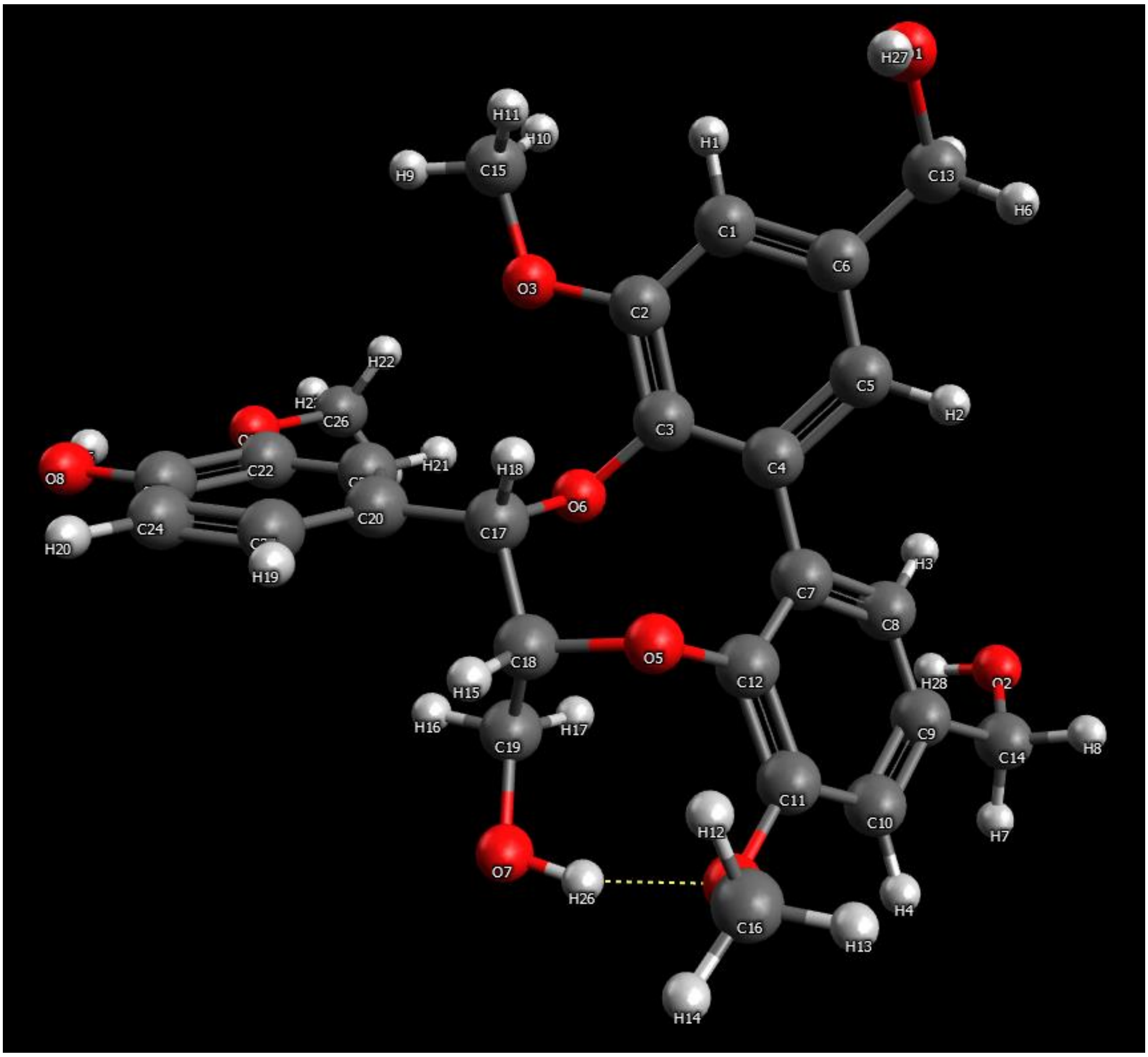

Figure 6 - Model of DBDO RS in the RS configuration. This means that hydrogens no. 15 and 18 have the same orientation – this configuration is also called ***cis****.*

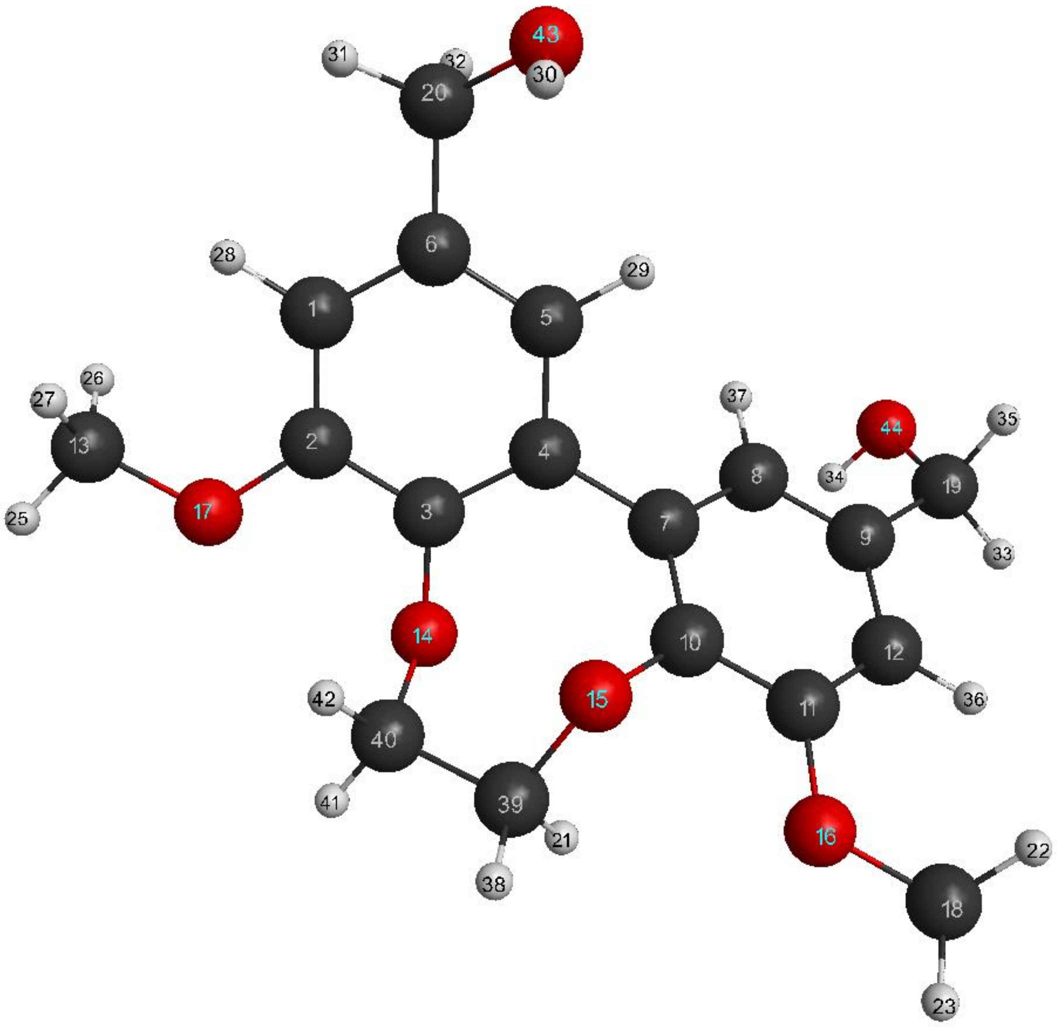

Figure 7 – DBDO backbone, which represents only the biphenyl part of DBDO. Calculation with Gamess.

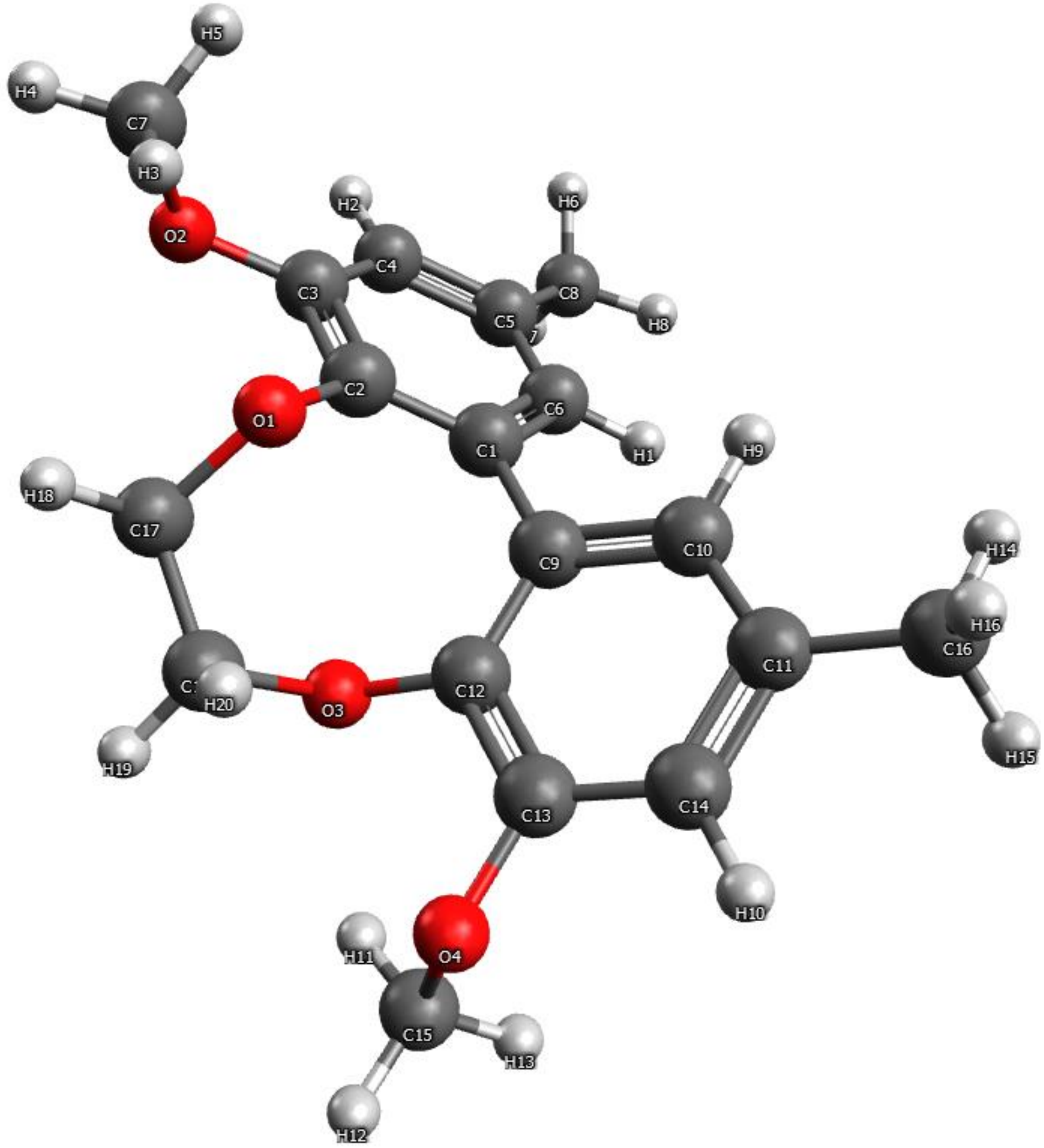

Figure 8 – DBDO – backbone, calculated structure. Interestingly, the methoxy groups are not in plane with the ring.

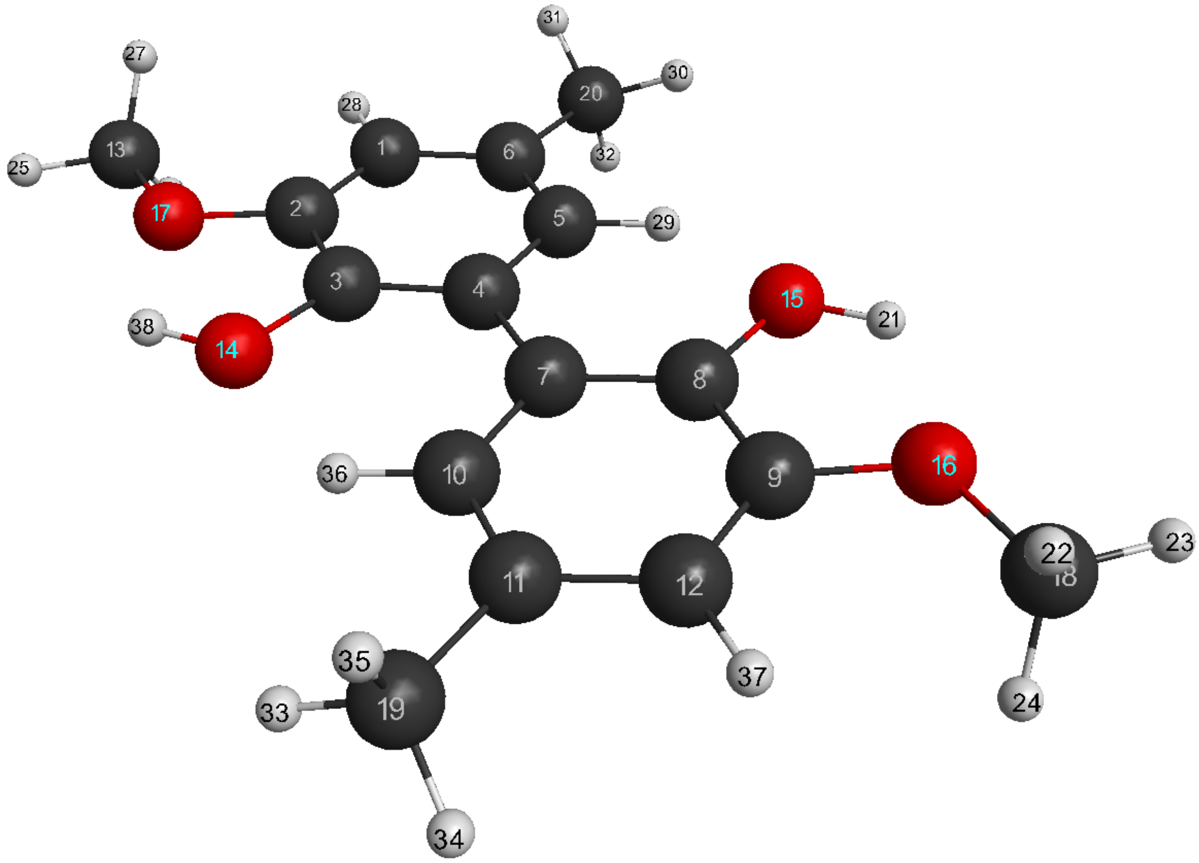

Figure 9 – Biphenyl unit 0°. The dihedral angle of the atoms 3, 4, 7, 10 is 4,38°.

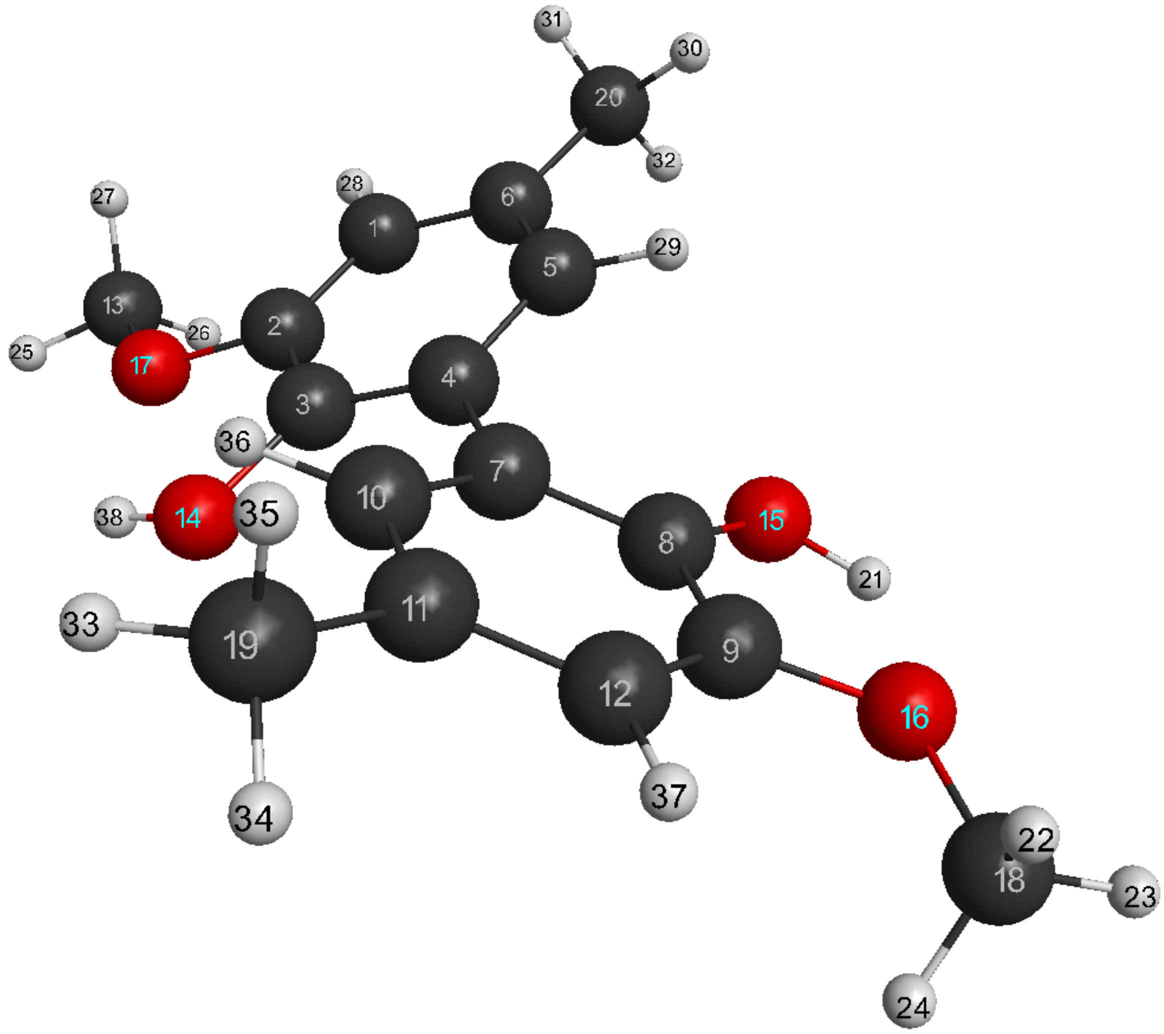

Figure 10 – Biphenyl unit 45°. The dihedral angle of atoms 3, 4, 7, 10 is 44,83°.

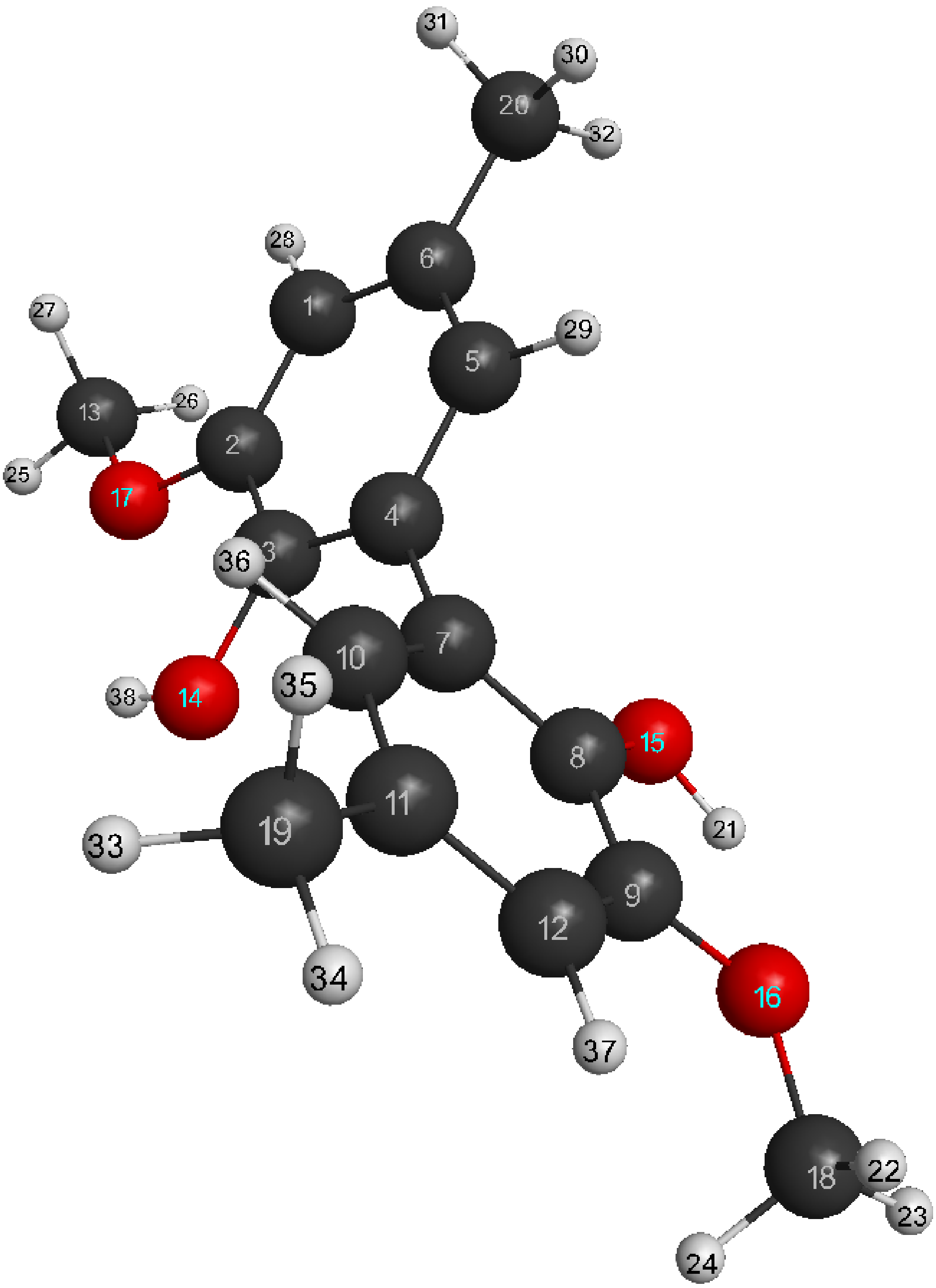

Figure 11 - Biphenyl unit 90°. The dihedral angle of atoms 3, 4, 7, 10 is 84,6°.

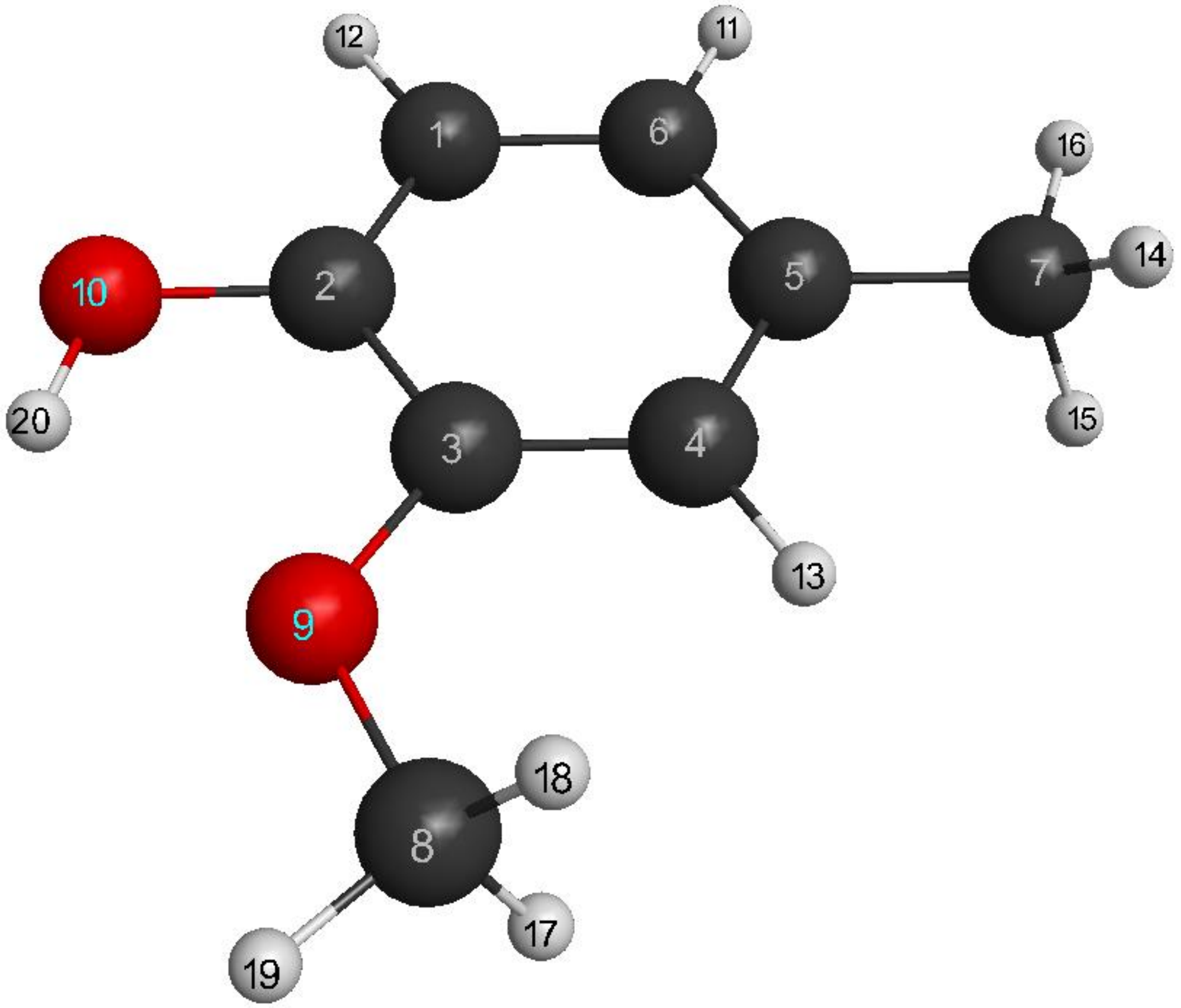

Figure 12 - Structure of the G-unit (2-methoxy-4-methylphenol). This compounds is the simplest G-unit and shows already most of the bands found also in bigger structures which are attributed to ring modes and is therefore a good model to understand asym-trisubstituted rings of the G-type. Vibrational spectra of compounds of this kind are explained in more detail in Bock and Gierlinger [6].

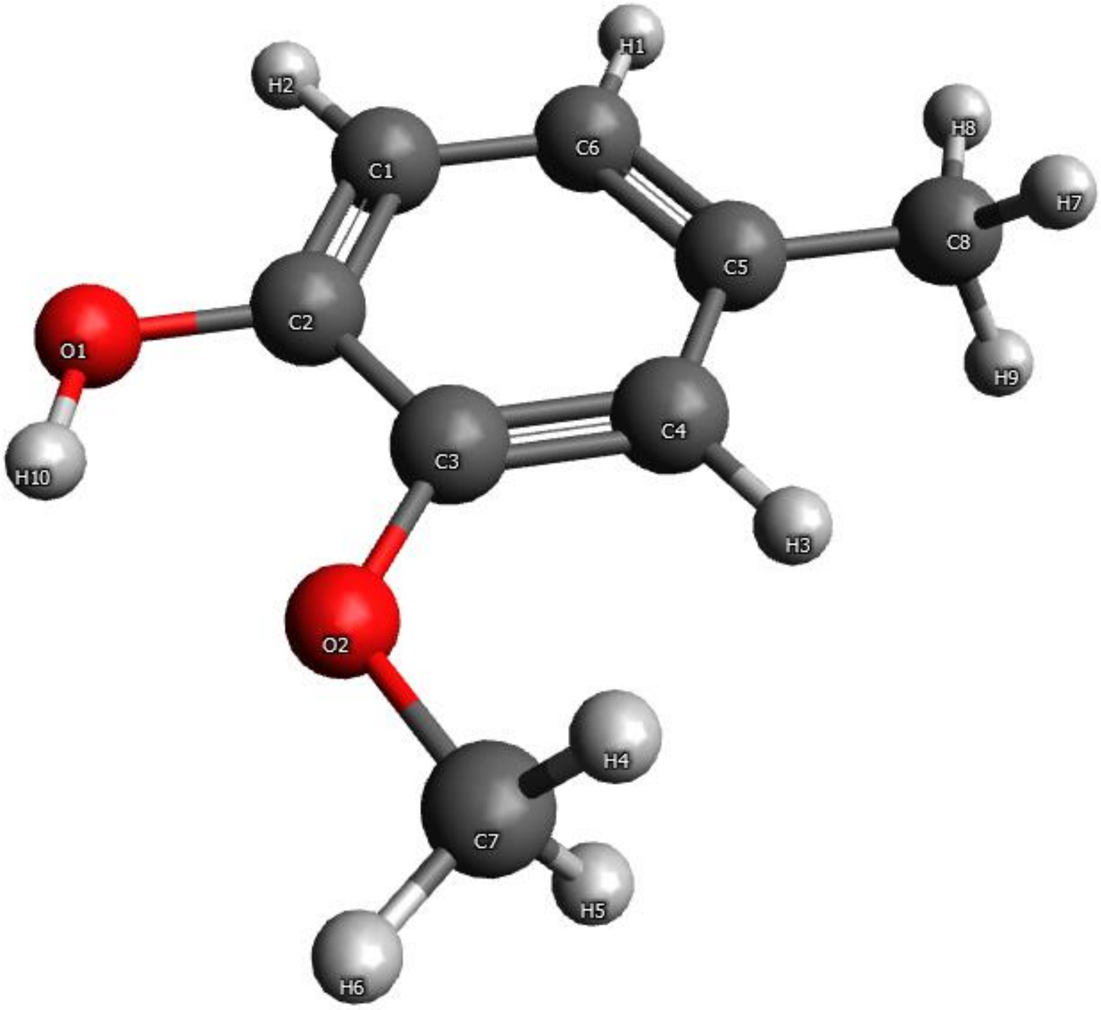

Figure 13 – Molecular model of the G-unit.

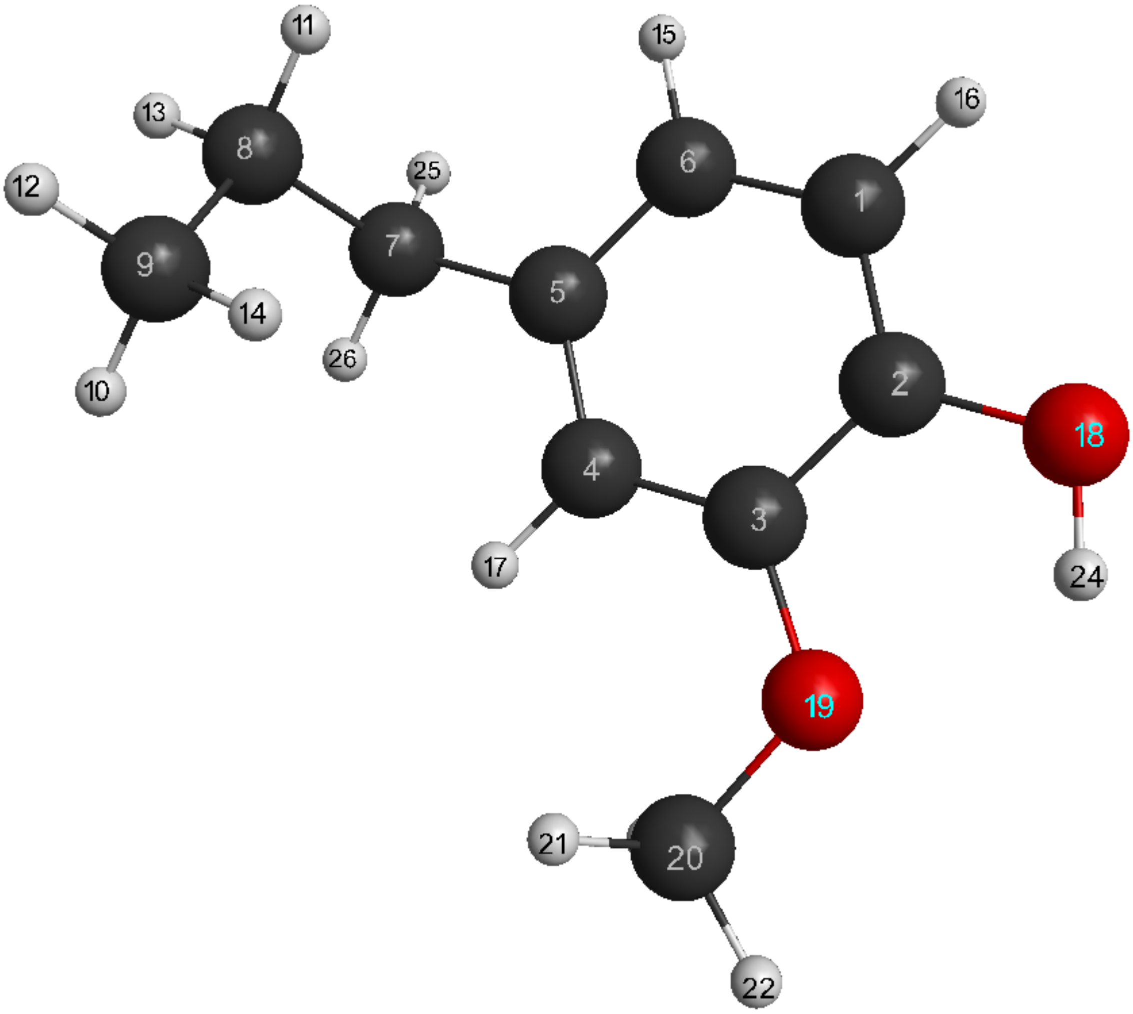

Figure 14 - Structure of 2-Methoxy-4-propylphenol used for the assignments. This compound resembles the coniferyl alcohol unit where the double bond (between C7 and C8) was used for polymerization. Except for the fact that the terminal OH-group on C9 is missing, it is an excellent model compound.

Infrared and Raman spectra of dibenzodioxocin

DBDO yields Raman spectra with little fluorescence. They are shown in Fig. 15.

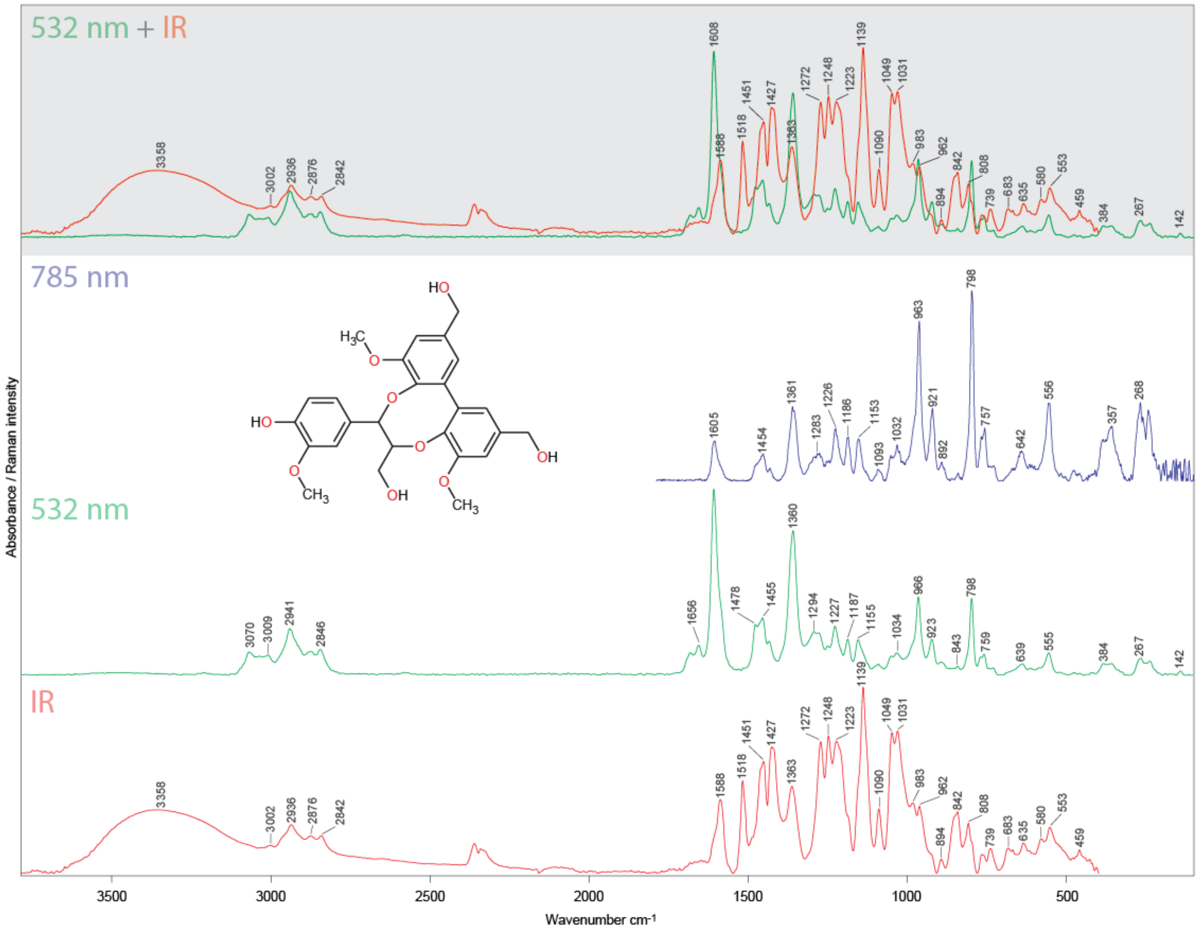

Figure 15 - Infrared and Raman spectra of DBDO. Shown is the overlay of Raman 532 with IR as well as the individual spectra. Spectra were cut and baseline corrected.

Raman spectra were recorded with different laser polarizations. Changing the laser polarization and recording scattering from every angle (no polarizers), the Raman spectra look very similar (Fig. 16, B). When the polarizers were set with respect to the incident laser polarization, slight changes were observed (spectra in Fig. 16, D).

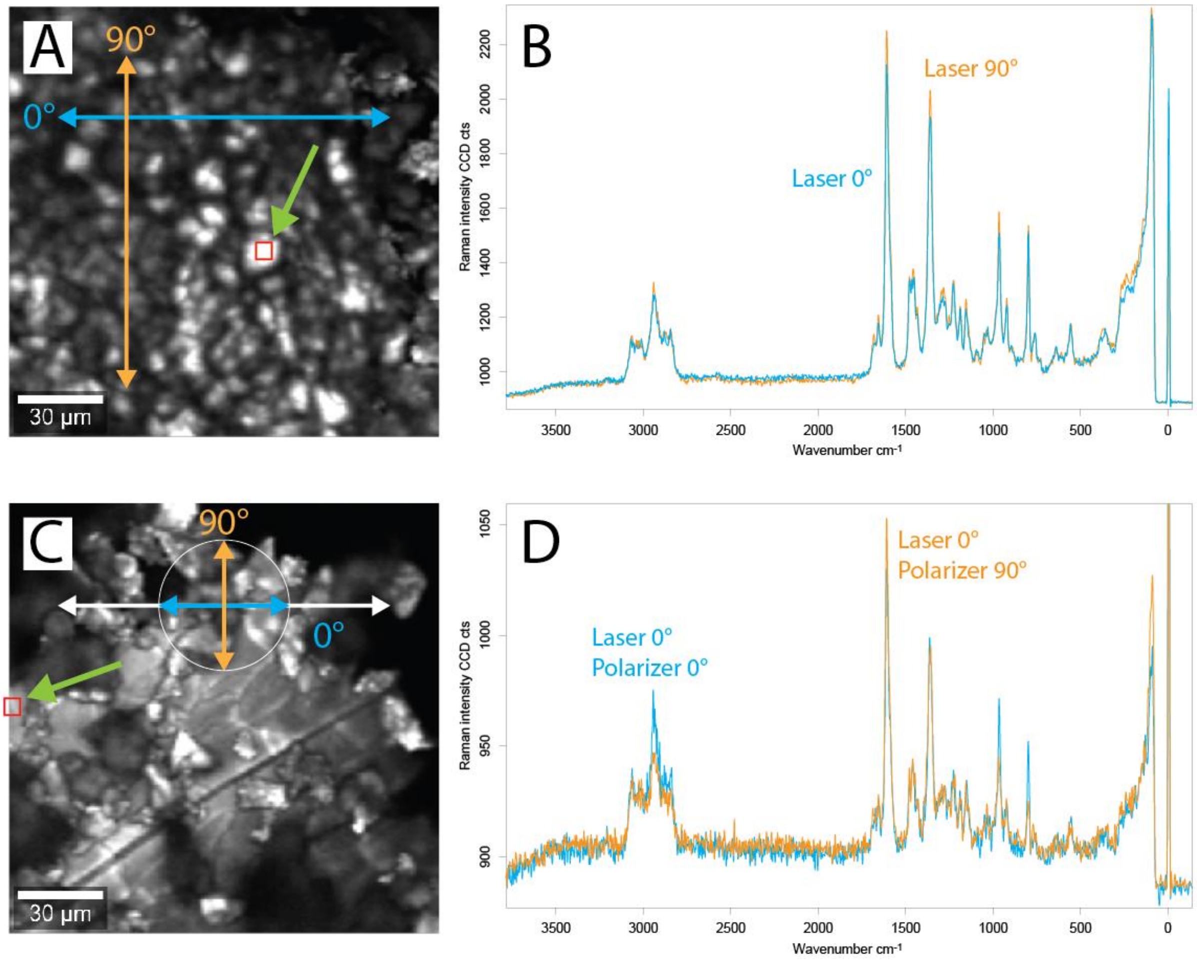

Figure 16 – Unprocessed, raw Raman 532 spectra of DBDO with different polarizations. ***A)*** *Raman intensity heat map (1600 cm^{-1}, 30 cm^{-1}width) of DBDO crystals. The laser polarization directions are indicated. The arrow points to the pixel where the spectra were taken from.* ***B)*** *Spectra taken from the red square in A). Almost no difference could be observed between the polarization directions.* ***C)*** *Heat map as in A), taken from a different position. While the laser polarization remained unchanged, the polarizers were either aligned (0°) with or perpendicular (90°) to the laser polarization. Spectra from a pixel (red square) are shown in* ***D)****. Three bands were found to differ: The symmetric CH_3 stretch (2941 cm^{-1}), ring mode 7a (966 cm^{-1}) and ring mode 1 (798 cm^{-1}).*

Temperature does not affect the Raman spectrum, only the fluorescence background is slightly increased, as shown in Fig. 17. This is contrary to the normal case, where a reduction in fluorescence would be expected, when a compound is heated up, because transitions to the ground state have lower probability. DBDO and other 5-5' structures (data not shown) show opposite behavior, this might be the reason for lignin to fluoresce at every temperature. Previous work saw the cause for fluorescence of 5-5' structures in the increased rotation of the biphenyl rings at elevated temperature.[7]

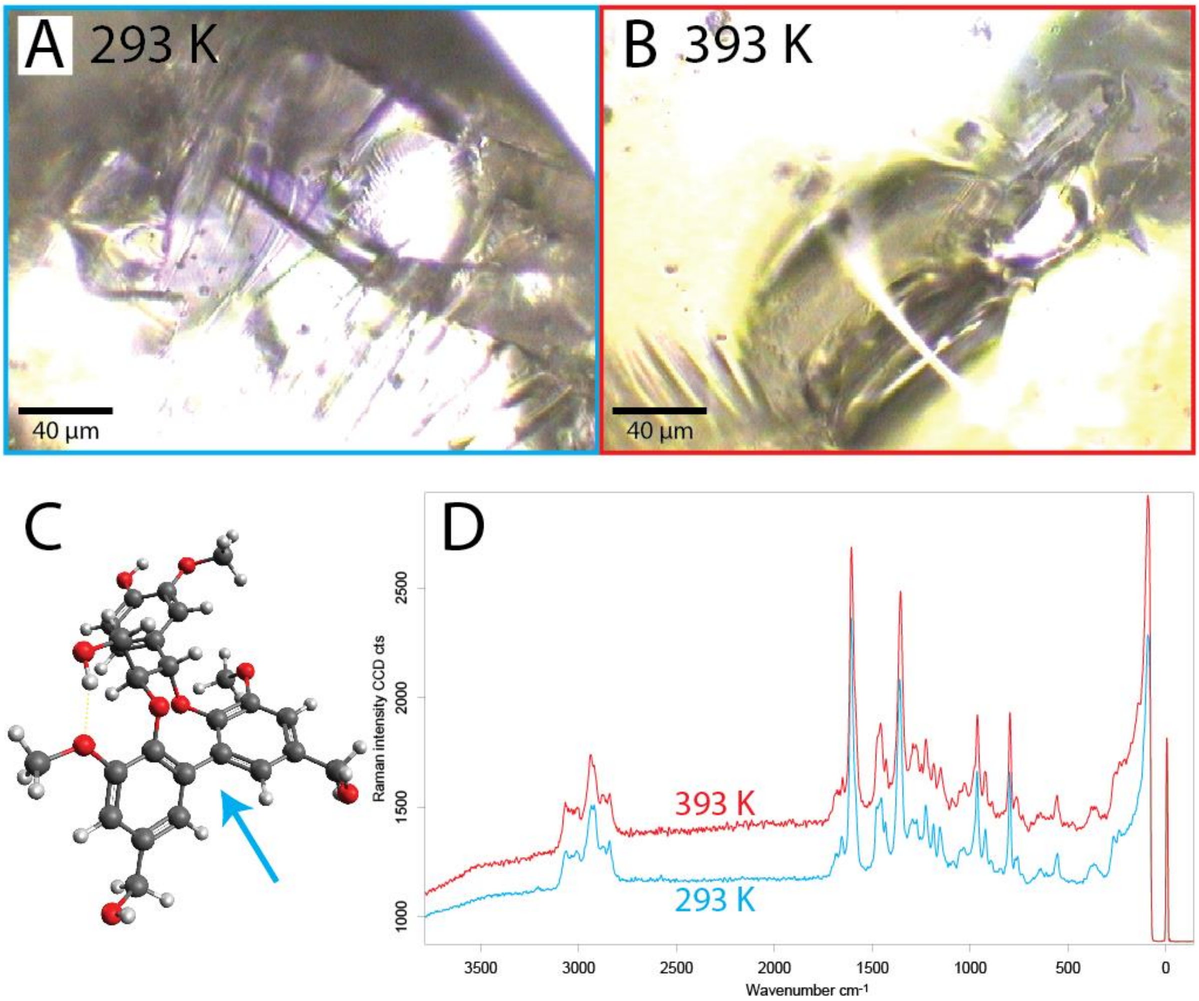

Figure 17 - Raman 532 spectra of DBDO at two temperatures. ***A)*** *Visual image of the crystal at 293K (20°C).* B) *Visual image of the substance at 393K (120°C). This was the limit of the heating stage. The crystals appear more glass-like, but did not really melt. Although TGA results (see Fig. 1) suggest some complex decomposition reaction, the Raman bands are still unaffected. This might be due to stronger scattering of DBDO in relation to decomposition products.* C) *Molecular structure of DBDO with the coannular bond marked. In DBDO, rotation of the biphenyl rings is hindered by the dioxocin linkage.* ***D)*** *Raman spectra of DBDO at 293 and 393K.*

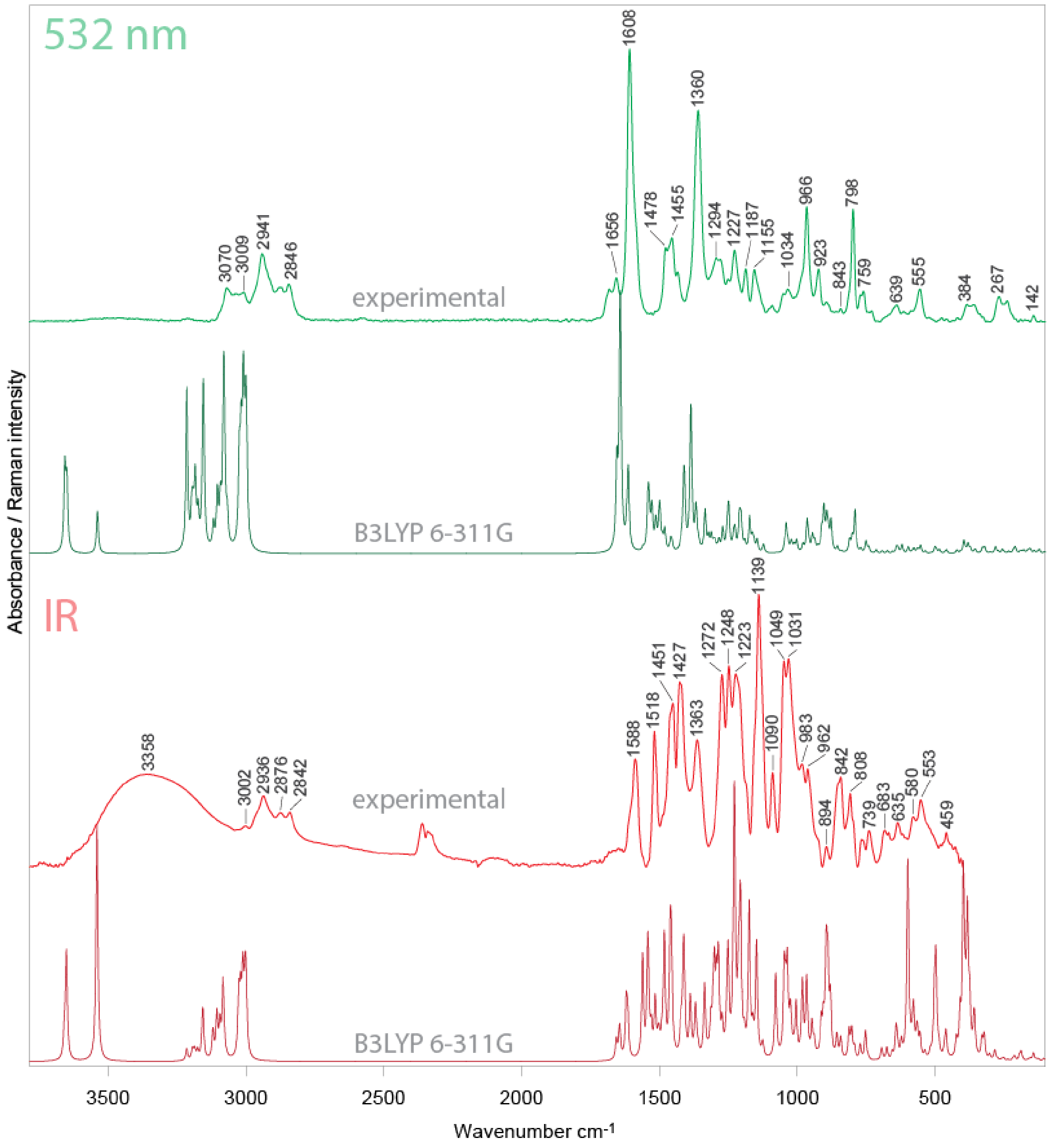

Figure 18 - Comparison of DBDO's experimental with calculated spectra. The latter were calculated with Gaussian. No scaling is applied in the figure.

Introductory notes on the vibrational analysis of biphenyls

DBDO consists of three G-rings, two of them are linked to each other via 5-5' bonding, resulting in a biphenyl unit. A coniferyl alcohol unit is linked to both rings in such a way that its double bond is opened up and linked to each of the biphenyl rings O4's.

From the vibrational point of view (see Fig. 19a), we deal with an uncoupled G-ring, a coupled S-ring and some simple CO, CC and CH oscillators. Taking into account that the aromatic ring is one of the most complicated groups in the group frequency approach, (e.g. in Fig. 19a) most of the bands will be caused by the aromatic nucleus[8]. The problem can therefore be reduced to an assignment problem of two differently substituted rings, which can explain most of the bands in the spectrum (we expect 30 G-ring and 60 S-ring bands, given that G and S will not couple).

It should be noted, that the so-called S-ring is not an S-ring as it is found in lignin, because the fourth substituent on the ring is not an oxygen but a carbon. Nevertheless, it is justified to treat this unit as an S-ring from the vibrational point of view, because it is identically substituted and this matters the most when dealing with vibrational modes of aromatic rings. The substituent change from O to C will of course cause frequency shifts and may affect couplings. The dipole moments may also differ, which affects the intensities in IR and Raman. In the remainder of the text, a single biphenyl ring is therefore treated as a S-unit.

The remaining CO, CC and CH oscillators of course have some contributions (for example the out-of-phase CO stretch of the methoxy groups, which causes a very strong IR band), but their frequencies do not change vary much between G and S rings, so that we can find them normally quite reliably. It should be noted that although the substitution of the biphenyl rings is not a real S-substitution, it is a asymmetric-tetrasubstitution of the ring, therefore the ring will behave in similar way as if position 5 was bearing an oxygen, because the substitution pattern has normally a bigger influence than the substituents (especially, if the mass change is not drastic, as is the case in going from O (16) to C (12). The coupling of the rings results in every mode appearing twice, in in-phase and out-of-phase combinations. Furthermore modes will be allowed to couple that normally won't do, because two rings are connected and this may shift their wavenumbers, so that modes come close enough to each other to interact. We therefore expect some modes to lose their vibrational character – which will be hard to identify in the computations.

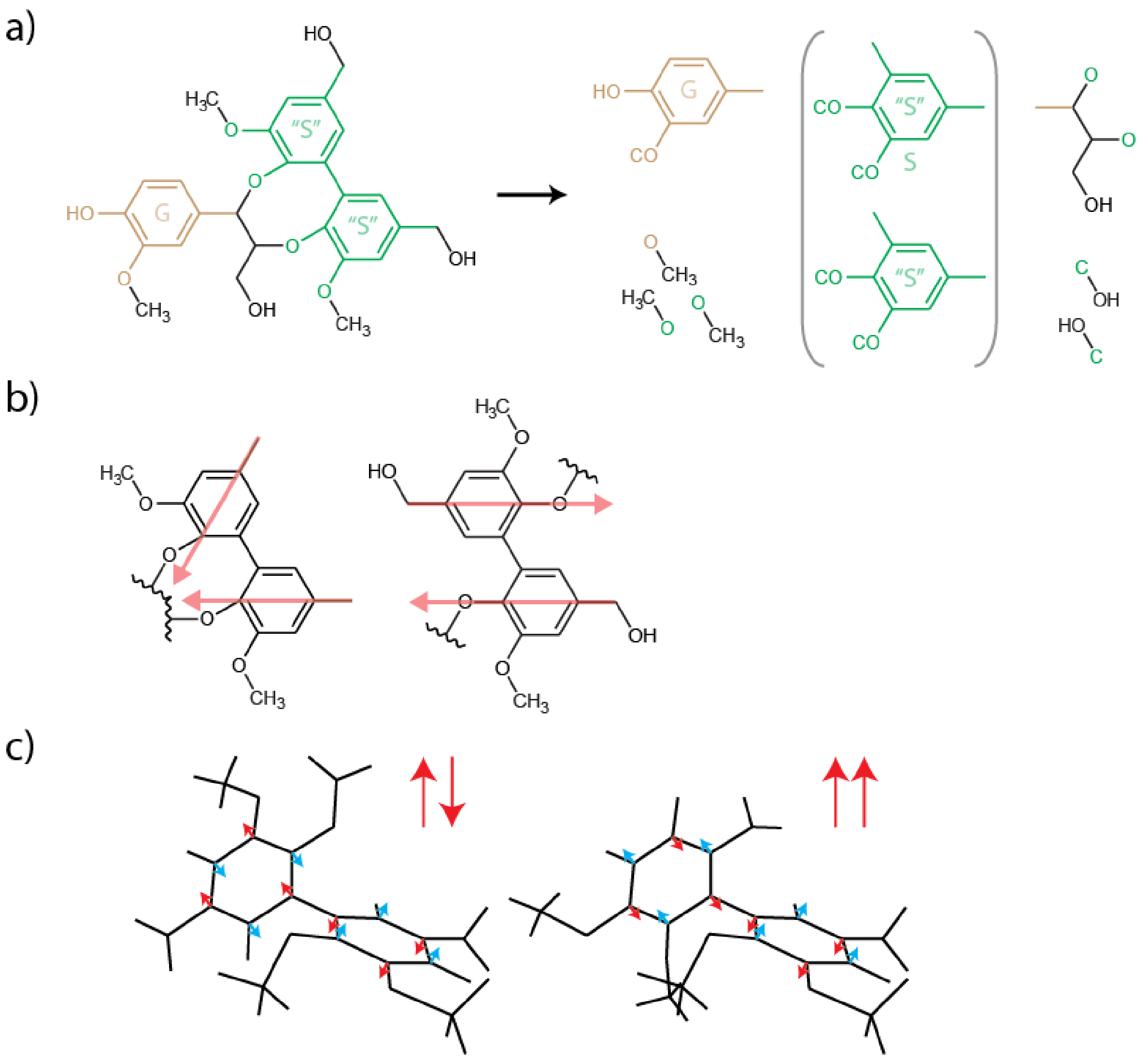

Figure 19 – a) Chemical structure of DBDO and how it can be separated for vibrational assignment purposes. The single ring of the biphenyl is treated as an S-unit, see text for details. b) Depending on the orientation of the two rings with respect to each other, individual dipole moments are enhanced or cancelled out. In-phase modes can therefore either exhibit strong or no infrared intensity, depending on the orientation. This is also true for the Raman intensity. c) Ring puckering of a biphenyl unit. If one ring becomes rotated by 180°, then the displacement pattern turns from an out-of-phase into an in-phase combination.

The bands of DBDO will now be assigned and commented in decreasing wavenumber order. DBDO is different from the other two molecules studied in that here both rings of the biphenyl unit are oriented the same way, whereas for DCAB and MCAB, they are orientated in opposite direction. This means that the activity of the in-phase and out-of-phase modes will differ theoretically between DBDO and DCAB/MCAB. Fig. 19b explains this in-phase combinations of a ring mode. The arrows indicate a change in dipole moment. It is clear that due to the different conformation, in DBDO a dipole moment will be created while in DCAB/MCAB it will cancel out. Although the pictorial representation is idealized, because neither of the moieties is planar, the computed results are in line with this consideration. Furthermore, also the frequency order might be changed upon conformational changes. Out-of-phase combinations are normally seen at higher wavenumbers (see also Colthup, Daly and Wiberley [8] for an extended discussion). However, this is true for the whole molecule. As can be seen in Fig. 19c, the in-phase combination of Φ4 is an out-of-phase displacement on molecular level, because both C_1 move in opposite directions. If the two rings were twisted

around the coannular bond by 180°, then it would be an in-phase displacement. The infrared and Raman activities change accordingly to this and that is why vibrational spectroscopy is also sensitive towards conformational changes.

Individual ring modes couple with each other, but this also depends on the angle between the two ring planes. It seems that angles of up to 45° still enable modes to couple with themselves as judged from unsubstituted biphenyl, which has an angle of 34-44°[9-11] When considering couplings of ring modes as in biphenyl units, normally we only talk about couplings of the same ring mode, i.e. mode 3 of ring A couples with mode 3 of ring B to give an in-phase and an out-of-phase combination. However, this is not the only possibility. In general, both rings in biphenyls have three possibilities:

a) The system is planar enough and both ring modes will couple to give in- and out-of-phase modes, i.e. mode 3 (ring A) couples with mode 3 (ring B) to give an in-phase 3 at frequency X and an out-of-phase 3 combination at frequency Y.
b) The system is not planar and each ring will perform its mode at a certain frequency. The other ring can remain stationary i.e. it only counters the movement to keep the center of mass unchanged, which means that mode 3 (ring A) will appear at frequency X and mode 3 (ring B) at frequency Y.
c) The system is not planar and each ring will perform its mode at a certain frequency, while the other ring performs another ring mode. For twisted system this means that one ring mode will be in-plane nuclear displacement and the other ring will have its atoms displaced out-of-plane, i.e. at mode 3 (ring A) and 16b (ring B) both appear at a certain frequency.

However, this option is only possible if there is a second ring mode in the vicinity of the first one that is close enough in frequency so that both rings can perform individual ring modes. In other words, these combinations can only happen in wavenumber regions below 1000 cm^{-1}, because the out-of-plane modes of S-rings appear in this interval.

This might be counter-intuitive from a symmetry viewpoint, as modes belonging to different symmetry species are not allowed to mix.[8] However, it should be remembered that molecular symmetry takes the whole molecule into consideration, which in our case, does not have symmetry elements other than the identity operation (C_1 point group). At the symmetry level of the individual benzene rings, it is therefore allowed to mix in-plane with out-of-plane modes.

Commented assignment of dibenzodioxocin

In table 2, the first column represents the Raman (532 nm) wavenumber, the second the IR wavenumber. The assignment is made by comparison with the previous work[6,8,12,13] as well as with our own computations and spectral library (parts of it are published in Felhofer, Prats-Mateu, Bock and Gierlinger [14] and Bock and Gierlinger [6]).

Φ [number]: denotes a ring mode in Wilson/Varsanyi notation.

DBDO: Dibenzodioxocin

BP: Biphenyl

ip: in-phase (used for mode relationships, i.e. two bonds stretch at the same time)

op: out-of-phase (i.e. one bond stretches while the other compresses)

*: Band only seen upon deconvolution

n.a.: Not available. Used for IR bands which could not be detected because of instrument cut-off

Explanatory notes on the C-H stretching modes

The fundamental work on the behavior of CH stretches of all kinds of compounds can be found in all standard publications dealing with vibrational spectroscopy. Although these enable the reader to understand the principal motions of these groups, detailed assignments in even simple molecules require more sophisticated approaches due to complicated coupling effects of some groups with their own overtones.

The standard quantum-chemical calculations applied to assignment problems of vibrational spectroscopy fail to calculate values close to the observed ones because of the aforementioned effects which need to be addressed by additional corrections.[15]

The assignments based on our B3LYP-functional, which is not corrected for anharmonicities are therefore the most uncertain in the CH stretch region. Nevertheless, a survey on the various types of CH groups and the effects which influence their frequencies can help to understand the vibrational spectrum.

We start hereby with counting the Hs in the molecule and assigning them to functional groups which are very familiar to chemists.

DBDO has 28 Hs, which can be separated into

- 9 Hs belonging to methoxy groups
- 6 Hs belonging to methylene groups
- 2 isolated Hs which do not have a second H on the same atom
- 7 Hs belonging to aromatic rings and
- 4 Hs belonging to OH groups which are not considered further

For the interpretation, this separation is useful and the reader should note that the grouping is based on the number of hydrogens which share a carbon and the type of that carbon, i.e. in a methyl group, three hydrogens share the same carbon and the two single hydrogens are different from ring Hs, which are also isolated, but their carbon is differently hybridized.

Furthermore, is drawn to the fact that the lone pair interactions can perturb C-H frequencies and that we have to also consider these effects also. It is well established[16], that lone electron pairs can interact with antibonding C-H orbitals, which will increase the bond length and decrease the frequency of these oscillators. In principle, it has been shown[16] that this back-donation can be applied to any orbitals of suitable symmetry and steric position, but let us consider here only the C-H bonds. DBDO possesses nine oxygen atoms, of which five are adjacent to carbons bearing hydrogens.

Let us now try to give some of the considerations necessary for assignment on the stretches of each of these groups in detail with the help of the aforementioned as well as standard publications.[8,17]

Methoxy groups

Methoxy groups are considered as methyl groups with an electronegative substituent. Therefore we can apply first the theory for methyl groups. The CH_3 group has three hydrogens which are chemically equal and share the same carbon, therefore their motions strongly couple. Under C_{3v} symmetry, this group will have one symmetric stretching mode and two asymmetric ones, which are degenerate. Furthermore, methyl groups are known for fermi resonances, where the first overtone of the symmetric bend interacts with the symmetric stretch[8,18]. The individual CH bonds in methoxy groups are no longer equal as the oxygen breaks the symmetry and one hydrogen is chemically different from the others. Methoxy groups show a distinct band at approximately 2850 cm^{-1} [19], however the reason for this is not clear from the literature. While the vibrational assignment to the symmetric stretch is consistent across all published work, earlier publications ascribe the lowering in frequency to a lone pair[†] – σ* interaction[16,18,25,26], while more recent work favors a fermi resonance with the bending overtone of the symmetric “umbrella” bending[15,27]. The implication is that this band will be present regardless of whether the oxygen lone pairs are involved in hyperconjugation with the aromatic p_z-orbitals or not, meaning that it does not matter for this mode whether the methoxy group is in-plane with the ring or rotated out. This can also be seen in molecules, where the methoxy group is forced out of the plane as in 2,6-dimethylanisole, which also has a band at 2864/2825 cm^{-1} (we are not certain on which of these is attributed to the methoxy group).[28]

Another consequence is that the methoxy group has two sets of hydrogens, a pair consisting of the hydrogens sticking out of the ring plane and a single hydrogen in-plane with the ring. Its modes are therefore better described as CH stretch, symmetric and anti-symmetric CH_2 stetch.[15]

In DBDO, the band at 2846 cm^{-1} is therefore assigned to the symmetric stretch of *all* methoxy groups.

The asymmetric stretches are less straight forward as they are not degenerate anymore and, as mentioned above and the displacement pattern shown in the textbook can no longer be applied. The in-plane asymmetric CH stretch (A’, considering the OMe group having C_s symmetry) is assigned to the higher wavenumber[28,29] (corresponds to the single CH stretch[15] – this makes sense from both the CH_3 and the CH + CH_2 arguments, as in this mode the isolated hydrogen has to counter the movement of the other two Hs with twice the amplitude) and the out-of-plane asymmetric stretch (corresponding to the anti-symmetric CH_2 stretch) is assigned to the lower wavenumber. Hence, by comparison with anisole, they are assigned to 3003 and to 2962 cm^{-1}, respectively.

[†] Note that the actual shape of the lone pair orbitals is of utter importance for this discussion. The VESPR model[20-22] was recently critically reviewed by Clauss et al.[23] and defended by Hiberty et al.[24] The conclusion was that both the VSEPR and the NBO theory can be used and there may be context-specific merits for preference of one over the other.

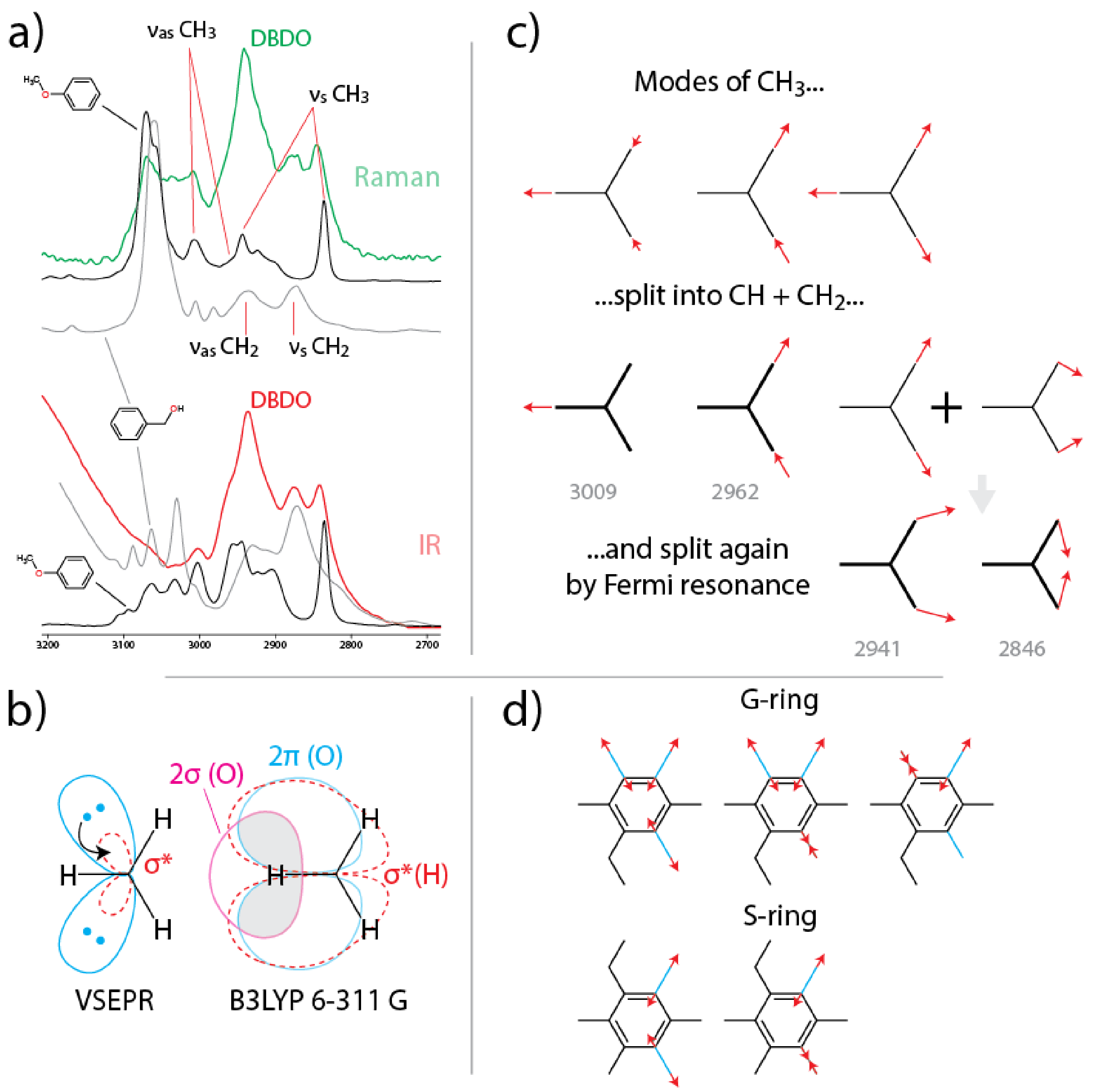

*Figure 20 – **a)** Raman and infrared spectra of DBDO compared to anisole and benzyl alcohol. **b)** The overlap of the oxygen lone pairs as seen by VSEPR theory and by a B3LYP calculation (Anisole, shown are HOMO -4, HOMO and LUMO +8). This shows that the initial explanation of the low frequency was justified as from the view of MO theory until it was replaced by the modern view that only Fermi resonance is responsible for the large split. **c)** CH stretching modes of the methyl group. The three modes according to group theory are split into a subset of a CH_2 group and a single H, because of influence of the adjacent oxygen. The symmetric CH_2 stretch further interacts with its own scissor bend to give the two modes shown here. **d)** The CH stretches of the two Ring systems involved.*

Methylene groups

Methylene groups are simple in terms of their stretching modes, because there are only two. DBDO has three CH_2 groups, and in all cases there is an OH group next to it. So in principle, the same reasoning, as for methoxy groups, can be invoked, namely that the hydrogens will become vibrationally decoupled because they interact with the oxygen lone pair and have their frequency downshifted. However, the oxygen can rotate freely around the CH_2-O bond and also participate in H-bonding, so here a sharp band is not expected. In Fig.20a, the spectra of benzyl alcohol are shown. Two broad bands are observed in the Raman spectrum, they correspond to the anti-symmetric (higher wavenumber) and symmetric stretching. We can assume that at the onset of the CH stretching region of DBDO there will be some contribution of those C-H oscillators, which are

interacting with the oxygen lone electron pairs as detailed above. This is in agreement with CH_2 groups adjacent to a nitrogen atom.[8]

By comparison with benzyl alcohol, it is apparent that the CH_2OH group causes bands at similar wavenumbers as the methoxy group. However the band at 2872 cm^{-1} is not present in anisole, so we assign the corresponding band in DBDO to the symmetric stretch of the methylene group. We further assume that the third methyl group will also come at this wavenumber.

Isolated hydrogens

There are two isolated hydrogens in DBDO, sitting next to each other on the carbons that are involved in the ether bridge to the biphenyl rings. The calculation shows them to be chemically equal to the extent that their stretching motions couple. The out-of-phase mode has a high calculated IR activity and the Raman activity of the in-phase stretch is rather low.

In 1,1-diphenyl propane, the CH lone stretch was assigned to 2889 cm^{-1}.[15]

Aromatic hydrogens

There are two differently substituted rings in DBDO, so there are two sets of hydrogen displacement patterns.

The G-ring has three hydrogens, two are adjacent to each other. The corresponding three CH stretching modes can be seen as the coupling product of the in-phase stretch of the H-pair with the lone H: (+ +) +; (+ +) - and (+ -) 0. They are assigned as modes 2, 20a and 20b, respecetively.

The S-ring has two hydrogens, therefore the combinations + + and + -, assigned as 2 and 20b, respectively. Considering the twist of the BP unit, it is not unreasonable to assume that the hydrogens next to the coannular bond exhibit a different environment and are therefore vibrationally decoupled from the other H on the same ring – according to the calculation, this also seems to be the case. Interestingly, the bending motions are coupled and can be described following the Varsany/Wilson modes.

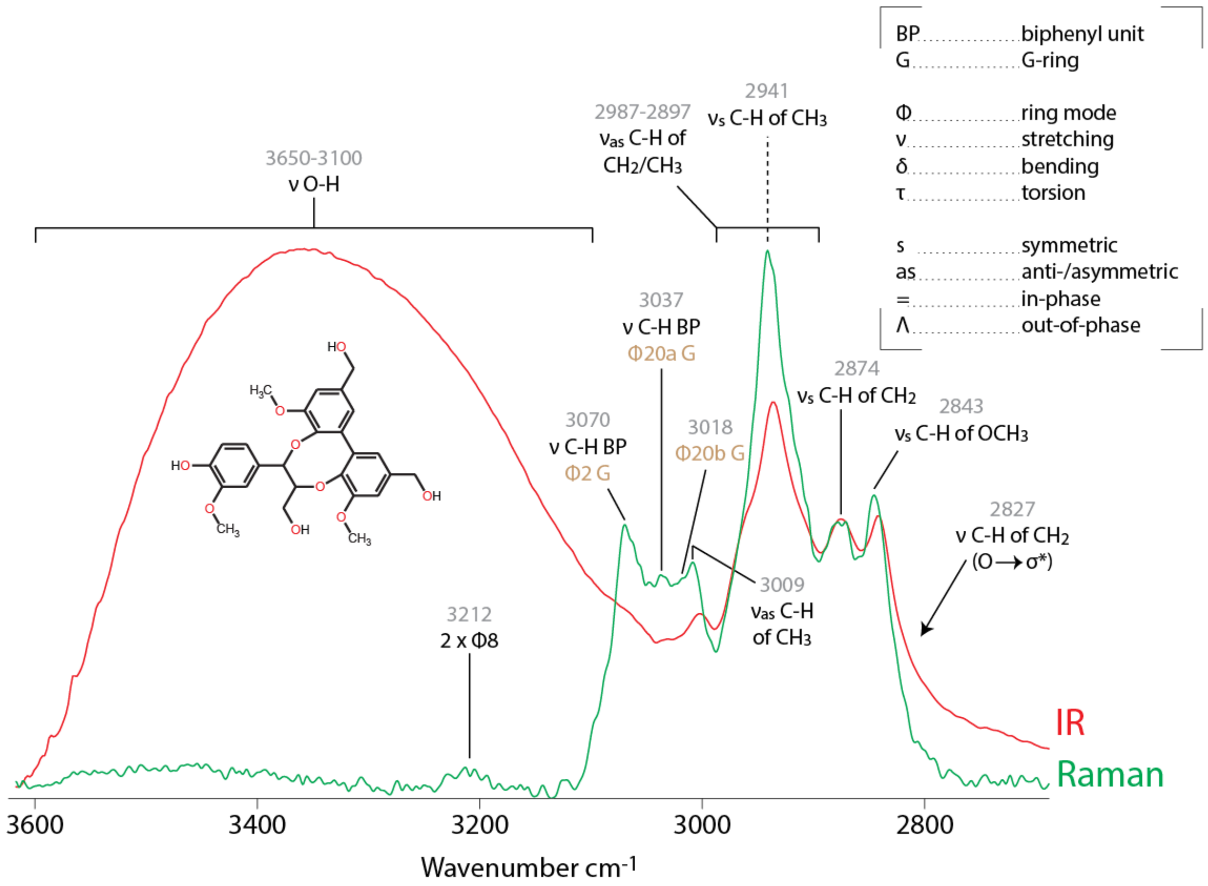

Figure 21 - Raman and IR spectra of DBDO, range 3600 -2650 cm^{-1}.

Table 2 - Assignment table of DBDO.

Raman	IR	Comment
3650-3300	3650-3100	OH stretch<br><br>Hydrogen bonding makes the band broad.
3212		First overtone of Φ8<br><br>This weak band is often seen in the Raman spectra of aromatic compounds.<br>1613 x 2 = 3226<br>1604 x 2 = 3208
3070		In-phase Φ2 of BP<br>Φ2 of G<br><br>A strong Raman band around 3065 cm^{-1} appears for all G- and S-ring compounds we measured, also mono-substituted rings have this band. The in-phase all-in-phase CH stretch of unsubstituted biphenyl is also observed at 3065 cm^{-1}, that is why we assign this bend to both G-ring and BP unit.
3037	3034*	CH stretching of rings of BP<br>Φ20a of G<br><br>By comparison with several G- and S-ring model compounds (2-methoxy-4-methylphenol, 2-methoxy-4-propylphenol, eugenol, eugenylacetate, 4-allyl-2,6-

		dimethoxyphenol, 4-methyl-2,6-dimethoxyphenol) it is apparent that all have two Raman bands at ~3065 and ~3010 cm^{-1} but no Raman band between these two peaks which reaches similar intensity than these two. Therefore, this band is attributed to the BP unit in the Raman.<br><br>In infrared, the band is not clearly resolved and will also contain the lone-H stretch of the G-ring.
3018		Φ20b of G
3009	3003	Asymmetric CH stretching of OCH_3<br><br>This is the mode which stretches in-plane of the ring and can be described also as single CH stretch of the C-H oscillator in-plane with the ring, directed towards the oxygen.
	2962*	Asymmetric CH stretching of OCH_3
2941	2936	Symmetric CH stretching of OCH_3<br>Anti-symmetric stretching of CH_2<br><br>In comparison with anisole and benzyl alcohol (see Fig. 20), it is clear, that this band has contributions of both compounds and therefore of both functional groups.<br>The symmetric CH stretching of the methoxy group is upshifted and split into two bands due to fermi-resonance with its own bending overtone.
2924	2910*	Asymmetric CH stretching of OCH_3<br>Anti-symmetric stretching of CH_2<br>Symmetric CH stretching of OCH_3<br><br>May also contain the in-phase stretch of the two isolated C-H oscillators.
2876	2874	Symmetric CH stretching of CH_2
2846	2843	Symmetric CH stretching of OCH_3<br><br>This mode is in fermi-resonance with its own symmetric bending at 1462/1451 cm^{-1}.
2827		CH stretch of CH_2<br><br>This is assigned to those CH stretches, where the σ*-orbital is donated by the adjacent oxygen’s lone electron pair.

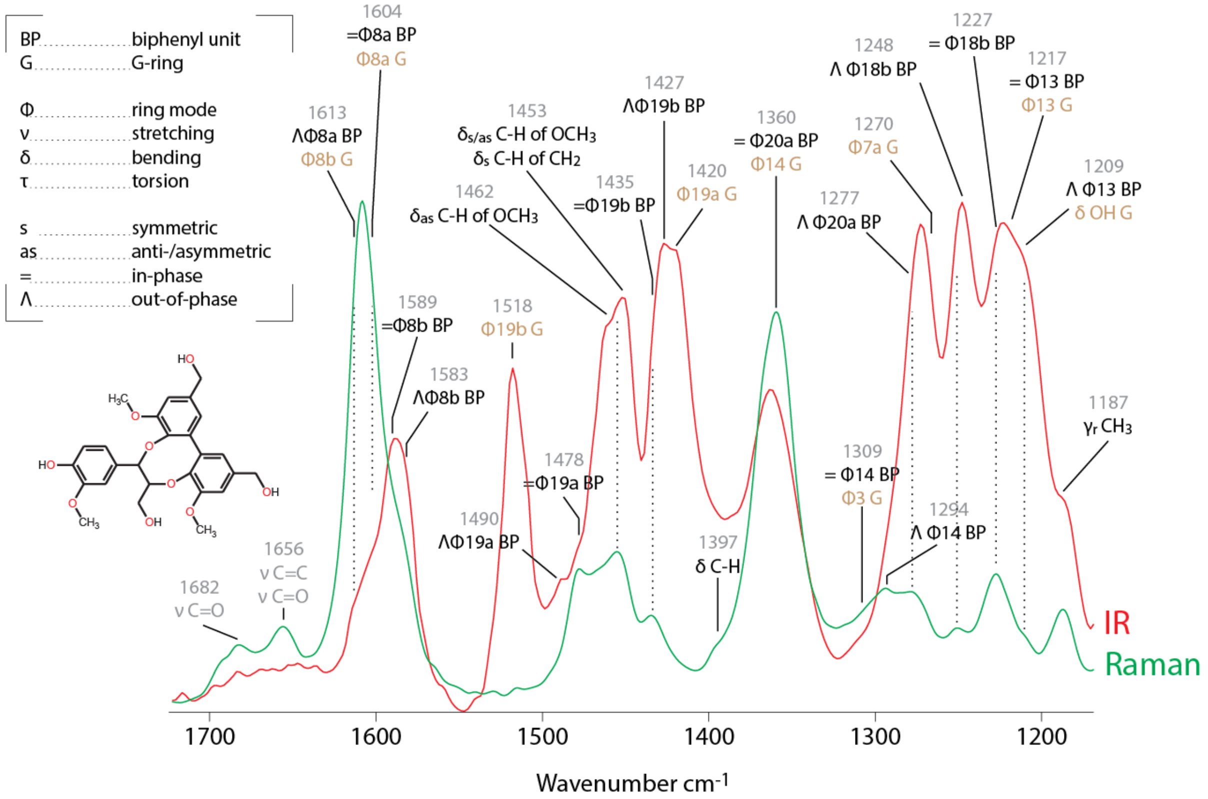

Figure 22 - Raman and IR spectra of DBDO, range 1700 -1200 cm⁻¹.

1682	1699	C=O stretch of impurity<br><br>Based on the lower wavenumber with respect to the reference value of carbonyls[17] and the rather strong Raman intensity, it is concluded that this carbonyl is in conjugation with another π-system. Since carbonyls of cinnamaldehydes appear normally at 1660 cm^{-1}, it could be the signal of alpha-bromo-ketone used in the synthesis (see Karkunen 1996[30] for details)
1656	1662	C=C stretch of coniferyl alcohol<br><br>Assigned to the conjugated C=C stretch of coniferyl alcohol, which was used in the synthesis.
1613*	1613*	Out-of-phase Φ8a of BP<br>Φ8b of G<br><br>The enhancement of vibration 8 upon conjugation is well known from the literature[31-34], although the enhancement effect seen in DBDO is attributed to resonance enhancement, because it is not observed at 785 nm. Since in unconjugated G-rings, Φ1 is in most of the cases stronger than Φ8, regardless of excitation wavelength, the Raman band is mainly attributed to the BP modes. The frequencies of both rings/modes are calculated close to each other. From frequency considerations it is also clear, that the G-ring should have both members of vibration 8 here, with 8b being at higher than 8a.[12]
1604*	1604*	In-phase Φ8a of BP<br>Φ8a of G
1593*	1589*	In-phase Φ8b of BP<br><br>Both in-phase and out-of-phase combination are calculated to have similar IR intensity. The Raman band derives mainly from the out-of-phase combination6

1585*	1583*	Out-of-phase Φ8b of BP
1515	1518	Φ19b of G<br><br>Assigned on the basis of 2-methoxy-4-methylphenol, 2-methoxy-4-propylphenol and eugenol.
	1488	Out-of-phase Φ19a of BP<br><br>Interestingly, the ring coupling downshifts this ring mode from the usual frequency of S-rings.
1478	1480	In-phase Φ19a of BP
1467		CH bending
1458	1462	CH bending of methoxy groups<br><br>The doublet at 1464/1453 is often observed in benzene rings bearing a methoxy group. Rings with more than one methoxy group can still possess these bands but they might become indistinct because additional bands resulting from coupling between the groups appear.<br>The symmetric bending (umbrella vibration) is upshifted in the presence of oxygen which makes both the asymmetric and symmetric bending of the methoxy group lie close together and which makes them difficult to distinguish.<br><br>The remaining eight hydrogens (three CH_2; H_{alpha} and H_{beta} should appear somewhere in this range but cannot be identified separately. The band is assigned to the methoxy groups because its shape is unchanged with respect to molecules which do not bear CH_2 groups. It is therefore assumed that the contribution, especially in the IR, is mainly from the methoxy groups.
1455	1451	CH bending of methoxy groups<br>CH bending of CH_2OH groups<br><br>Benzyl alcohol shows a sharp band at 1454 which can only be due to the CH_2 bend. It is therefore likely, that this band contains contribution of the CH_2OH groups.
1435		In-phase Φ19b of BP<br><br>Assigned because of the medium Raman band which agrees with the computed Raman activity of this mode.
	1427	Out-of-phase Φ19b of BP
	1420	Φ19a of G<br><br>Assigned to this band because the corresponding Raman intensity is the lowest and this mode is expected to have the lowest Raman activity in comparison to the 19b combinations of the BP unit.
1397*	1397*	CH bending<br><br>According to the computation, this is mainly the wagging of the CH_2OH groups and bending of the alpha (H37) and beta-Hs (H32, see Fig.4).
1389*	1386*	CH bending<br><br>See above.

Explanatory notes on the C-X stretching and C-H bending modes

The following part of the spectrum may look difficult from the amount of modes calculated, but with vibrational theory of the ring we can work through it. The modes encountered here are mainly CH bendings of the ring and CX stretches (X=Substituent). There are two hydrogens per S-ring and three per G-ring. Assuming that the S-ring hydrogens will couple, we arrive at four modes for the biphenyl unit, these are + + + + (ip Φ18b), + + - - (op Φ18b), + - - + (op Φ18a) and + - + - (ip Φ18a). The G-ring has three modes (3, 18b, 15). Without interaction with aliphatic CH bendings, there will be seven modes in total for ring CHs. Likewise, the CX oscillators couple with ring modes, but there are only as many couplings above 1000 cm^{-1} as there are radial benzene modes around 1000 cm^{-1}. The other CX modes will couple to the radial bending modes of vibration 6 and therefore be found below 1000 cm^{-1} [12]. For this, the G- and S-rings have to be considered separately. The coupling patterns for the CX stretches with the S-ring are the same like shown for the CH bendings. It is not clear, however, which C-X stretch will couple with a ring mode and therefore the pictorial representation in Fig. 23 can only serve an illustrative purpose. While the modes 20a and 13 can be unambiguously assigned, our computations give different coupling patterns for the C-X stretches with the ring modes 6a and 6b for the three different compounds, and it can be assumed that the conformation will have an effect on this. This means that sometimes it is the substituent on position 4, which couples to the ring mode, sometimes it is the substituent 3 or 5 or combinations of them. If all ring modes are consumed, the remaining substituents (see also Fig. 25) will only be counteracted by the ring, but do not couple to it. Regarding modes 6a and 6b, it is clear that the substituents in position 3 and 5 of the ring can in principle couple with both modes, whereas the substituents in position 1 and 4 can only couple with mode 6a. Mode 6b is therefore considered to be the one coupling with the methoxy groups and 6a can couple with one or both substituents in positions 1 and 4.

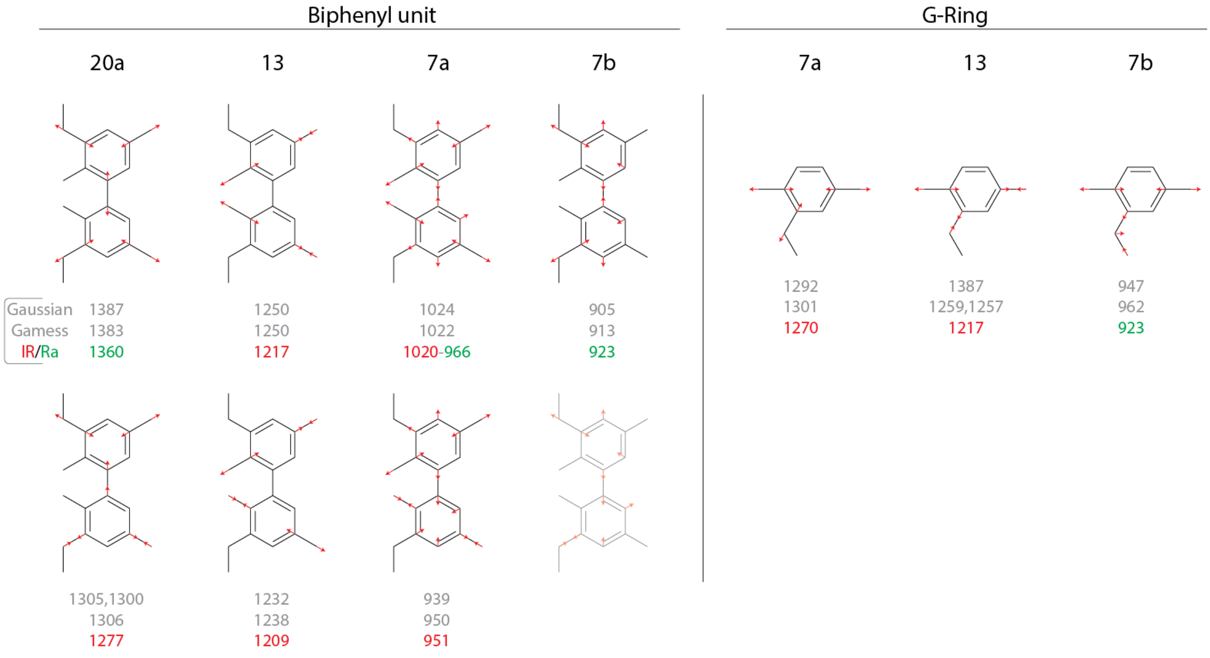

Figure 23 – The general coupling pattern of substituents with the ring in lignin 5-5'-structures and in G-rings. The calculated frequencies are compared with the experimental assignments. Only the modes classified as substituent stretches are shown. Note that the substituent stretches will be denoted as CH-stretching modes, the respective in-phase combinations (substituent to ring) will be the modes 1 for 20a, 12 for 13, 6a for 7a and 6b for 7b. Mode 13 is shown in the displacement pattern often calculated – only the carbons in position 1 and 4 are moving. The third C-X oscillator participates only little in this motion. The biphenyl unit has not 8, but only 7 substituents, because both rings share the coannular bond. One phase combination is therefore missing – the calculation showed that this will be 7b. Couplings of substituent groups are not shown, some can be seen in Fig. 25.

Furthermore, in the BP, both rings are coupled with each other, which will duplicate the number of modes and for every ring mode there will be an in-phase and out-of-phase combination observed, if

there is no other mode that can interact with it (in such cases, additional splits occur as we are dealing with a coupling-cascade and it gets more and more complicated to find all modes).

Two modes are observed at stable frequencies over all single G-rings we measured so far, so that we can confidently assign them. These are the bands at ~1035 cm^{-1} and ~920 cm^{-1}.

1360	1363	In-phase Φ20a of BP<br><br>The vibrational form of this mode does not really follow its description, because it is mainly the heavy loaded triangle of the ring which is moving. That is why we additionally denote this mode by the symbol ▲. It shows breathing character and is therefore normally strong in Raman.
1307*	1309*	In-phase Φ14 of BP<br>Φ3 of G<br>Aliphatic CH bending
1294	1293*	Out-of-phase Φ14 of BP
1279	1277*	Out-of-phase 20b of BP
	1270*	Φ7a of G<br><br>The assignment was made by comparing with 2-methoxy-4-methylphenol, 2-methoxy-4-propylphenol and eugenol.
1251	1248	Out-of-phase Φ18b of BP<br>Aliphatic CH bending<br><br>By comparison with 2-methoxy-4-methylphenol, 2-methoxy-4-propylphenol and eugenol, it is clear that neither of them possesses this band. That is why it is assigned to the BP unit in accordance with the computations.<br><br>The aliphatic CH bending must also appear somewhere in this band complex but is computed (and expected) to make only a small contribution to the spectrum. These modes are likely hidden and not resolved.
1227	1226*	In-phase Φ18b of BP<br>Φ13 G<br>Aliphatic CH bending<br><br>The Raman spectrum seems to display mainly the BP mode, because this has a high computed Raman activity.<br>By contrast, in the IR spectrum most of the contribution probably comes from the G-ring as ring vibration 13 normally has strong infrared intensity. The in-phase Ring-H-bending is computed to not change the dipole moment much.
1220*	1217*	In-phase Φ13 of BP
1210*	1209*	Out-of-phase Φ13 of BP;<br>OH bending;<br>CH rocking of CH_2OH

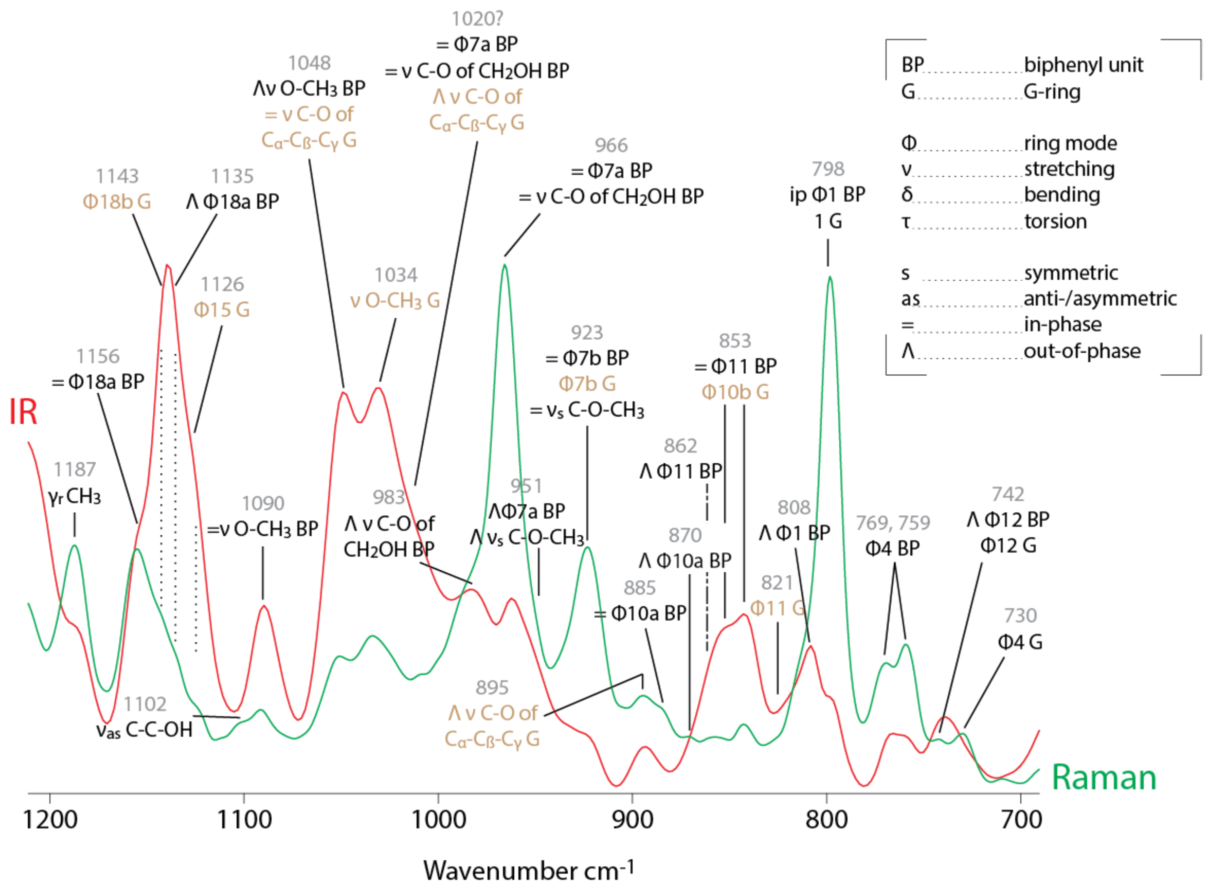

Figure 24 - Raman and IR spectra of DBDO, range 1200 -700 cm^{-1}.

1187	1185	CH rocking of methoxy groups
1155	1156*	In-phase Φ18a of BP<br><br>Φ18b of G-rings come a little bit lower (see next band) and also the calculations for DBDO show in-phase Φ18a of BP to have a very high Raman activity, therefore the assignment is chosen.
1143*	1139	Φ18b of G<br><br>Comparison with 2-methoxy-4-methylphenol, 2-methoxy-4-propylphenol and eugenol shows that ring modes 18b and 15 of G-rings seem to be rather stable in terms of wavenumber regardless of the ring substitution in position 4, if the extended substituent does not contain a double bond.
1135*		Out-of-phase Φ18a of BP
1123*	1126*	Φ15 of G

Explanatory notes on the C-O stretches

There are fourteen C-O oscillators in the molecule, eight of them do not involve an aromatic carbon – these are: 3x Methoxy C-O, 2x CH_2-OH of BP, 3x C-O of C_α-C_β-C_γ. This means that these eight do not necessarily have to couple to ring modes, so they are expected in the normal range of C-O oscillators: roughly 1200 – 800 cm^{-1}.[8] However, from symmetry considerations, there are two pairs of similar oscillators: the methoxy groups of the BP and the alcohol endgroup C-OHs of BP. Furthermore, also the tail of the coniferyl alcohol unit *can* be viewed as one unit with three C-O oscillators. In this case, three coupling patterns should emerge (+ + +), (+ 0 -) and (+ - +), which is also supported from the calculation.

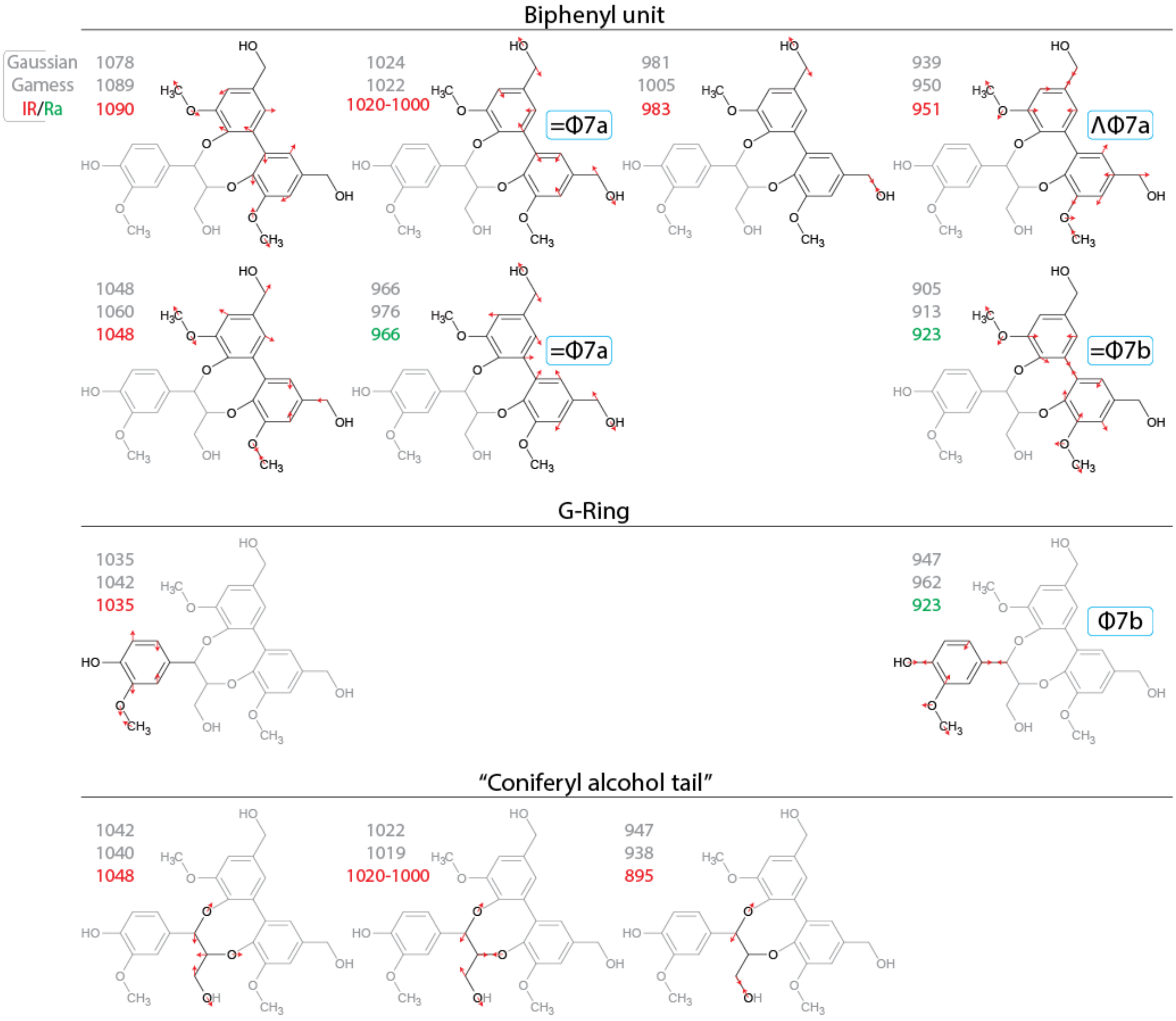

Figure 25 - C-O stretching modes in the region 1100 - 900 cm-1. Ring modes are indicated. Note, that the in-phase Φ7a of BP is split into two modes as it couples with the symmetric C-O stretch. Out-of-phase Φ7b is missing, because the rings share two carbons with each other, so not every C-X mode exists. The permutations can be carried out in the same way for bendings also.

Additionally, two ring modes fall into this region which normally couple with methoxy groups – 7a and 7b (G only 7b). There are three rings, so there should be five modes (7b G, 2x 7a BP, 2x 7b BP). However, the BP ring modes will couple, so that there are two BP combinations for each ring plus the single G-ring mode. Interestingly, the calculations for DBDO (both Gamess and Gaussian) show that there arise higher level combinations, as ring combinations couple with C-O oscillator combinations.

1102		Anti-symmetric stretch of the $C_{ß}$-C_{α}-OH group<br><br>Assignment based on ethanol, cinnamyl-, coniferyl-, sinapyl- and o-methoxycinnamyl alcohol. Also the computations show these carbons to stretch at roughly this wavenumber. Despite the motion is localized in the 8-membered ring of DBDO, this band still cannot be regarded as indicative for such unit, because the aforementioned alcohols also possess it.
1091	1090	In-phase stretch of the O-CH_3 groups of BP
1052	1048	Out-of-phase stretch of the O-CH_3 groups of BP<br>Symmetric stretch of the C-O oscillators of the coniferyl alcohol tail<br><br>This band is common to all three BPs.
1032	1034	CO stretching of methoxy group of G

		Assigned by comparing with simple G-molecules, which all have their CO stretches around 1035 cm^{-1}. Matches well with the calculation.
1020-1000	1020-1000	There are bands hidden between the bands 1031 cm^{-1} and 983 cm^{-1} and according to the calculation, there should be the following modes:<br><br>CO stretching of $C_α$-$C_ß$-$C_γ$<br>Out-of-phase (In-phase Φ7a of BP) (In-phase C-O of CH_2OH BP)<br><br>The calculation shows two strong infrared bands between the methoxy CO stretchtes and the BP CO stretches. By comparing with coniferyl alcohol and 3,4,5-ttrimethoxybenzylalcohol, one calculated mode is accepted, this is the CO stretch of the tail of the coniferyl alcohol unit connected to the BP unit. The bond length change of the C-OH bond is calculated to have the highest contribution to this normal mode, although the other C-O oscillators connected to $C_α$ and $C_ß$ also move in-phase, so that this mode could also be described as the in-phase C-O stretching mode of the $C_α$-$C_ß$-$C_γ$.<br><br>The other mode is computed to be a "coupling of a coupling" of a mode. The CO stretches of the CH_2OH groups couple to give two modes (see Fig.25), where one of them, the symmetric one, couples again with the in-phase combination of ring mode 7a of the BP unit, which by itself is a coupling of the two individual ring modes. This results in two modes, one where the in-phase 7a BP ring mode couples out-of-phase with the in-phase CO stretching of the CH_2OH group, the other where the coupling is in-phase – they are calculated to 1024 cm^{-1} and 966 cm^{-1}, respectively. This is seen clearly by the movement of the ring carbons 1,5,8 and 10, which, in the in-phase mode, radially move out of the ring when the C-O oscillator stretches, whereas in the out-of-phase combination the ring carbons move in during the stretching of the CO bond.
985	983	Out-of-phase CO stretching of CH_2OH of BP
966	962	In-phase (In-phase Φ7a of BP) (In-phase C-O of CH_2OH BP)<br><br>In this mode, the C-O oscillators stretch in-phase with the ring carbons.
949*	951*	Out-of-phase Φ7a of BP<br>Out-of-phase symmetric C-O-C stretching of the biphenyl methoxy groups<br><br>Normally out-of-phase relationships of substituent stretches at positions 3 and 5 of asym-tetrasubstituted rings are coupled to ring mode 7b. From the displacement pattern calculated for DBDO, it seems that mode 7a also couples to the out-of-phase symmetric C-O-C stretching of the methoxy groups.
923	921*	In-phase Φ7b of BP<br>Φ7b of G<br>Symmetric C-O-C stretching of the three methoxy groups
895	894	Out-of-phase CO stretching of $C_α$-$C_ß$-$C_γ$
888		In-phase Φ10a of BP
871	870*	Out-of-phase Φ10a of BP<br><br>Based on the assumption that this mode will create a small dipole moment change, which could be at the onset of this large peak.
860*	862*	Out-of-phase Φ11 of BP

		This mode is calculated to have only a medium dipole moment change. Furthermore, it is an out-of-phase combination. Both arguments together make it feasible to set this mode to the shoulder of the out-of-plane-CH-band-complex.
853*	854	In-phase Φ11 of BP<br>Φ10b of G<br><br>There are two strong peaks which probably derive from the in-phase umbrella motion of the aromatic hydrogens of the BP unit, because this mode is expected to create a large change in dipole moment. However, at around this wavenumber also mode 10b of the G-ring is observed in simpler model compounds. We therefore decided to assign this and the following band to a combination mode of the in-phase mode 11 of BP together with 10b of the G-ring. From experience, the in-phase coupling should be the one at lower wavenumber, however the accompanying Raman band is stronger so that we think this could be the mode, where the in-phase umbrella of the BP is countered by 10b of the G-ring. Therefore, this band is assigned to the all-in-phase-umbrella mode of the molecule.
843	842	In-phase Φ11 of BP<br>Φ10b of G<br><br>Based on the Raman activity, this band is assigned to the combination, where the in-phase umbrella of the BP unit moves out-of-phase with the lone-H of the G-ring.
	823*	Φ11 of G
	816*	
	808	Out-of-phase Φ1 of BP<br><br>The displacement modes, where the substituents move in the same direction as the ring carbons are located for G- and S-rings in the 800 – 500 cm^{-1}. These modes are denoted as Φ1 and Φ12. For asym-tri and asym-tetrasubstitution, they cannot be unequivocally distinguished anymore and it is therefore conventional to assign the displacement of the less-loaded triangle to Φ12 and the heavy-loaded triangle to Φ1.[12] However, our computations on several G- and S-rings suggest, that a better description of the actual displacements would be achieved by reversing that order. This means that the light-loaded triangle of the ring will be denoted as Φ1.<br><br>Furthermore, the computations show that these modes can interact with mode 4, which results in a combination mode, where the ring carbons also move out of the plane in the breathing modes.<br>While computations of the BP subunit alone show both modes to interact less, meaning that their original nature is still discernible; in DBDO strong mixing is present. It is also possible that one ring performs an in-plane mode while the other ring is in an out-of-plane mode, as discussed above. Given this, we assign this band to the out-of-phase combination of ring stretching, because looking at G- and S-rings in general, we assume that this is the domain of Φ1.<br>The out-of-phase combination is not expected to change the polarizability much, but will, if the rings are twisted, create a change in dipole moment, because O1 and O3 (see Fig.19) will move in the same direction.

		The band is therefore assigned to mode 1, although there might be partial mixing with mode 4 or combinations of each ring performing a different mode might be possible.
799	798	In-phase Φ1 of BP<br>Φ1 of G<br><br>Following the same argument as above and noting that this band is one of the strongest Raman bands, it is assigned to the in-phase ring stretching of the BP and the G-ring.<br>Interestingly, this mode is calculated to be much lower for the isolated BP-units (727 cm^{-1}; Gau-BP-unit; 739 cm^{-1} Gms-BP-unit); in DBDO this mode can no longer be clearly identified.<br><br>Part of this band is Φ1 of the G-ring. This mode normally has good Raman intensity as well and is relatively stable at 790 cm^{-1} (2-methoxy-4-methylphenol: 789; 2-methoxy-4-propylphenol: 794 cm^{-1}; Eugenol: 793 cm^{-1}).
769	766	In-phase Φ4 of BP<br><br>This and the following band stick out in DBDO by comparing it to spectra of G-rings – they are higher in wavenumber than typically expected for mode 12 in G-rings and the shape of the doublet is similar to the doublet next to it (IR: 807, 798 cm^{-1}). It is therefore likely that these doublet represents both combinations of mode 4 of the BP – the assignment of this band to the in-phase combination is based on the stronger IR intensity than that of band 758 cm^{-1}. It is to be noted that according to the calculations this band also involves mode 12 of the G-ring, which we assign to the lower band complex at 731 cm^{-1}.
759	758	Out-of-phase Φ4 of BP
742	739	Out-of-phase Φ12 of BP<br>Φ12 of G<br><br>This is an interesting example on how tightly interacting modes alter the frequency. Mode 12 is normally found around 580 cm^{-1}, but since both rings are connected in a way that they share a common atom (the H of one is the C of the other), both modes can interact and will fall apart. The separation is about 500 cm^{-1}! Such huge frequency splits are rare, the normal separation based on our model compounds and calculations is not higher than 30 cm^{-1} as a rule of thumb.<br><br>Based on 2-methoxy-4-propylphenol, this band is also expected to have contribution from Φ12 of the G-ring.
730		Φ4 of G

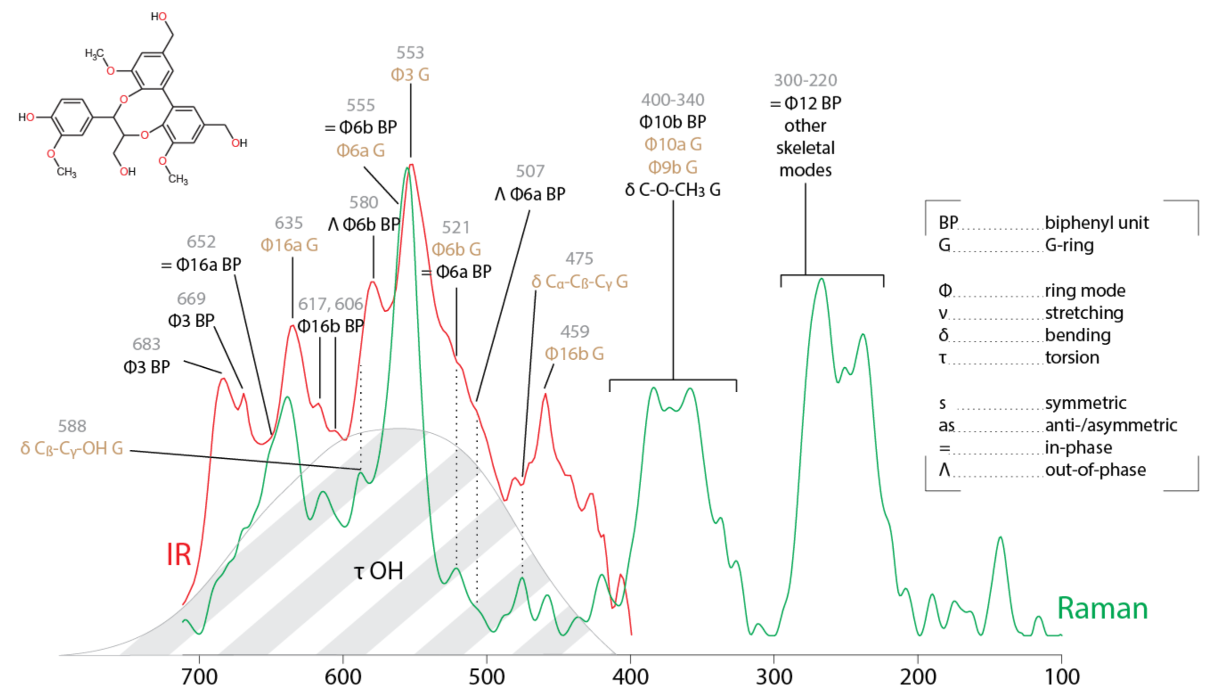

Figure 26 - Raman and IR spectra of DBDO, range 700 -100 cm⁻¹. A broad OH-torsion band is underlying part of this region in the IR – it is shown only for illustrative purposes – the actual shape might differ!

Bands from here on get increasingly difficult to assign, because often the whole molecule is involved in a vibrational mode. Additionally, ring modes lose their characteristic displacement patterns and acquire mixed in-plane/out-of-plane forms. The whole region is underlaid with a broad absorption which derives from the hindered OH rotation (=torsion). The assignment is here heavily based on comparing the spectra with reference compounds, since the computational results are often contradictory to one another.

Our infrared data are only available down to 400 cm^{-1}. Below one can make only an educated guess.

674	683	Φ3 of BP
	669	Φ3 of BP
652		In-phase Φ16a of BP<br><br>Assigned to the in-phase combination based on the high Raman intensity compared with lower IR intensity.
639	635	Out-of-phase Φ16a<br>Φ16a of G
614	617	In-phase Φ16b of BP<br><br>As with mode Φ16a, both modes are close together. This band is assigned to the in-phase combination based on Raman intensity considerations (as with Φ16a)
	606	Out-of-phase Φ16b of BP
588		
	580	Out-of-phase Φ6b of BP
555	553	In-phase Φ6b of BP<br>Φ6a of G<br>Φ3 of G

		Interesting case where the IR and the Raman band show different modes of the molecule. The in-phase radial skeletal mode of the biphenyl unit is expected to have strong Raman intensity – which is also shown by the calculations. The calculated infrared activity is medium, probably because of the directional movement of the coannular bond. In contrast, ring mode 3 of G-units normally does not show much Raman intensity, but it can be identified often in the IR spectra. The calculations predict a medium band for this mode.
521		In-phase Φ6a of BP<br>Φ6b of G
507		Out-of-phase Φ6a of BP
	480	δ C_α-C_β-C_γ of G
458	459	Φ16b of G
400-340	n.a.	δ C-O-CH_3<br>Φ10b of BP<br>Φ10a of G<br>Φ9a of G<br><br>This and the following band are always observed in G- and S-rings. They normally give good Raman intensity and consist of two modes and are often so close that only one band can be observed in simple model compounds.<br><br>One component is the bending of the C-O-CH_3 group. In G-rings, this mode often gives a medium line; in S-rings it is often among the strongest bands. This is due to coupling with the second group, which increases the polarizability for the in-phase combination. Calculations show that in G-rings this mode can further interact with bending of the other substituents, and the specific pattern would be described as ring mode 9a.<br><br>A hindered ring rotation (10a in G, 10b in S) often accompanies this line, but is normally weaker, especially in S-rings. Sometimes its wavenumber is higher, sometimes lower than the methoxy bending.
300-220	n.a.	In-phase Φ12 of BP<br><br>As already noted above (see Raman band 731), tight interaction causes the combinations of ring mode 12 of the BP unit to split by hundreds of wavenumbers. The displacement pattern of this mode is best described as a translational movement of both rings against each other. This conversion of a ring mode to a skeletal mode of the molecule is also an explanation why this combination appears at a very low wavenumber. This also is also seen in the parent molecule (biphenyl) which has its in-phase combination of the ring stretching at 328 cm^{-1}.<br>Note that this mode is the counterpart to the strongly Raman-active mode at 1361 cm^{-1} in a sense as here both substituent and ring carbon move in-phase with respect to each other, whereas the band at 1361 cm^{-1} resembles the out-of-phase combination.
142	n.a.	

Nuclear magnetic resonance (NMR) spectra of dibenzodioxocin

NMR-spectra of DBDO are shown in Fig. 27 and 28. The peak shifts are tabulated in Tab.3 and 4.

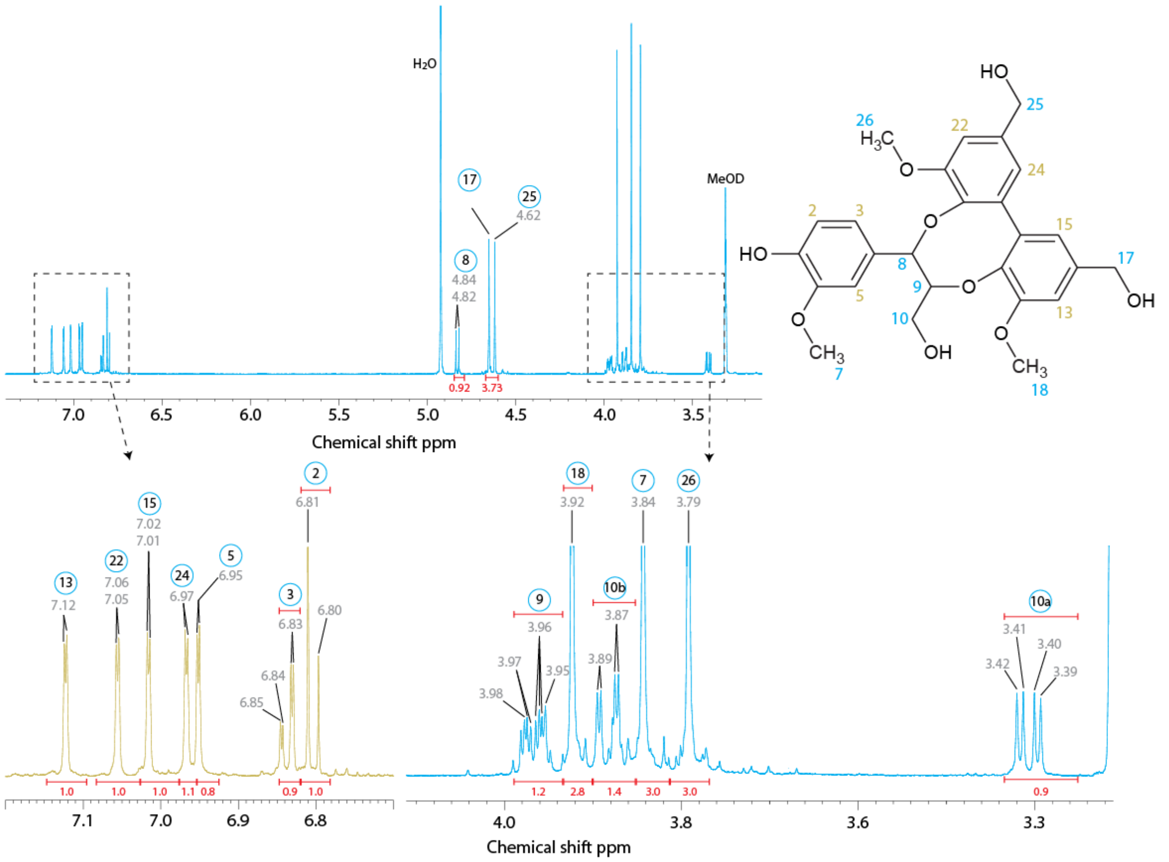

Figure 27 – ^{1}H-NMR spectra of DBDO. Measured in MeOD.

Atom	H shift	Multiplicity	J	Integral
10a	3.4064	dd	11.9, 4.2	
26	3.7917	s		3
7	3.8429	s		3
10b	3.8829	dd	11.9, 2.4	1
18	3.9234	s		3
9	3.9674	ddd	10.1, 4.3, 2.4	1
25	4.6158	s		2
17	4.6486	s		2
8	4.8273	d	10.1	1
2	6.8034	d	8.0	1
3	6.8374	dd	8.2, 1.8	1
5	6.9516	d	1.8	1
24	6.9669	d	1.9	1

15	7.0157	d	1.9	1
22	7.0556	d	1.9	1
13	7.1226	d	1.9	1

Table 3 – [1]H-shifts, multiplicities, J-coupling and integral values of DBDO measured in MeOD. The atom numbers correspond to the numbers in Fig. 27.

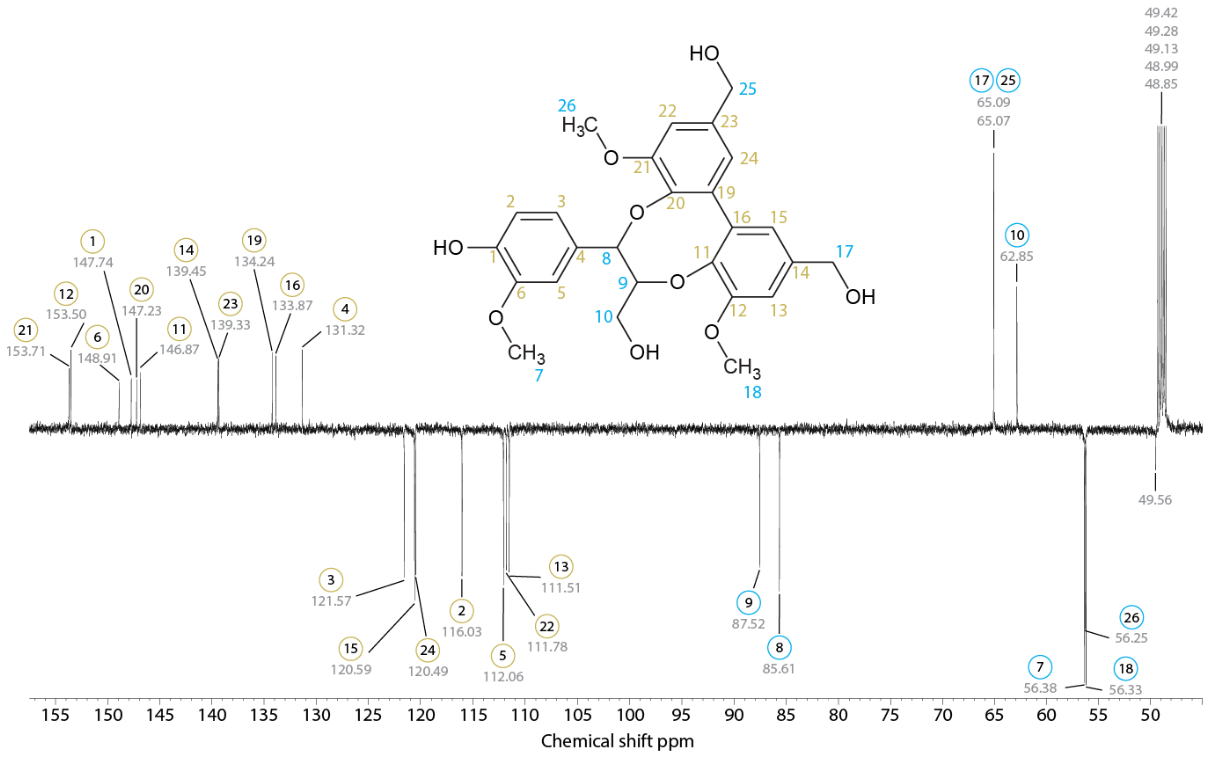

Figure 28 - ^{13}C spectra of DBDO.

C shift	Atom number
26	56.2495
18	56.3279
7	56.3810
10	62.8527
25	65.0720
17	65.0944
8	85.6093
9	87.5172
13	111.5053
22	111.7827
5	112.0613
2	116.0334
24	120.4926
15	120.5850
3	121.5749
4	131.3247
16	133.8694
19	134.2414

23	139.3293
14	139.4500
11	146.8703
20	147.2275
1	147.7384
6	148.9093
12	153.4971
21	153.7099

Table 4 – ^{13}C shifts of DBDO measured in MeOD. The atom numbers correspond to the those shown in Fig. 28.

UV-Vis spectrum of DBDO

Fig. 29 shows the UV-Vis spectrum of DBDO measured in ethanol.

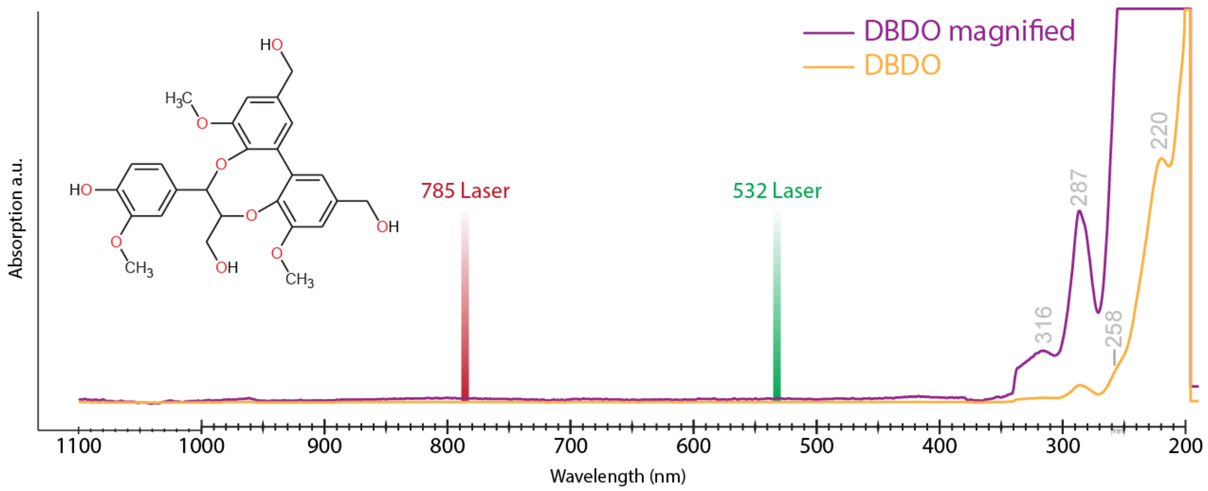

Figure 29 - UV-Vis spectrum DBDO. The violett spectrum is the magnification of the orange one. Excitation wavelengths of the lasers used in the experiments are also shown.

Vibrational modes of substituted benzene rings

Vibrational modes of the benzene ring are substitution-sensitive. Both wavenumber and intensity will be affected by the substituent(s). Fig. 30 shows the notation for G-rings, this is the coniferyl alcohol moiety joined over its ß and γ carbon. Fig. 31 shows the notation for S-rings, this can be applied to the biphenyl rings (see “Introductory notes on the vibrational analysis of biphenyls”).

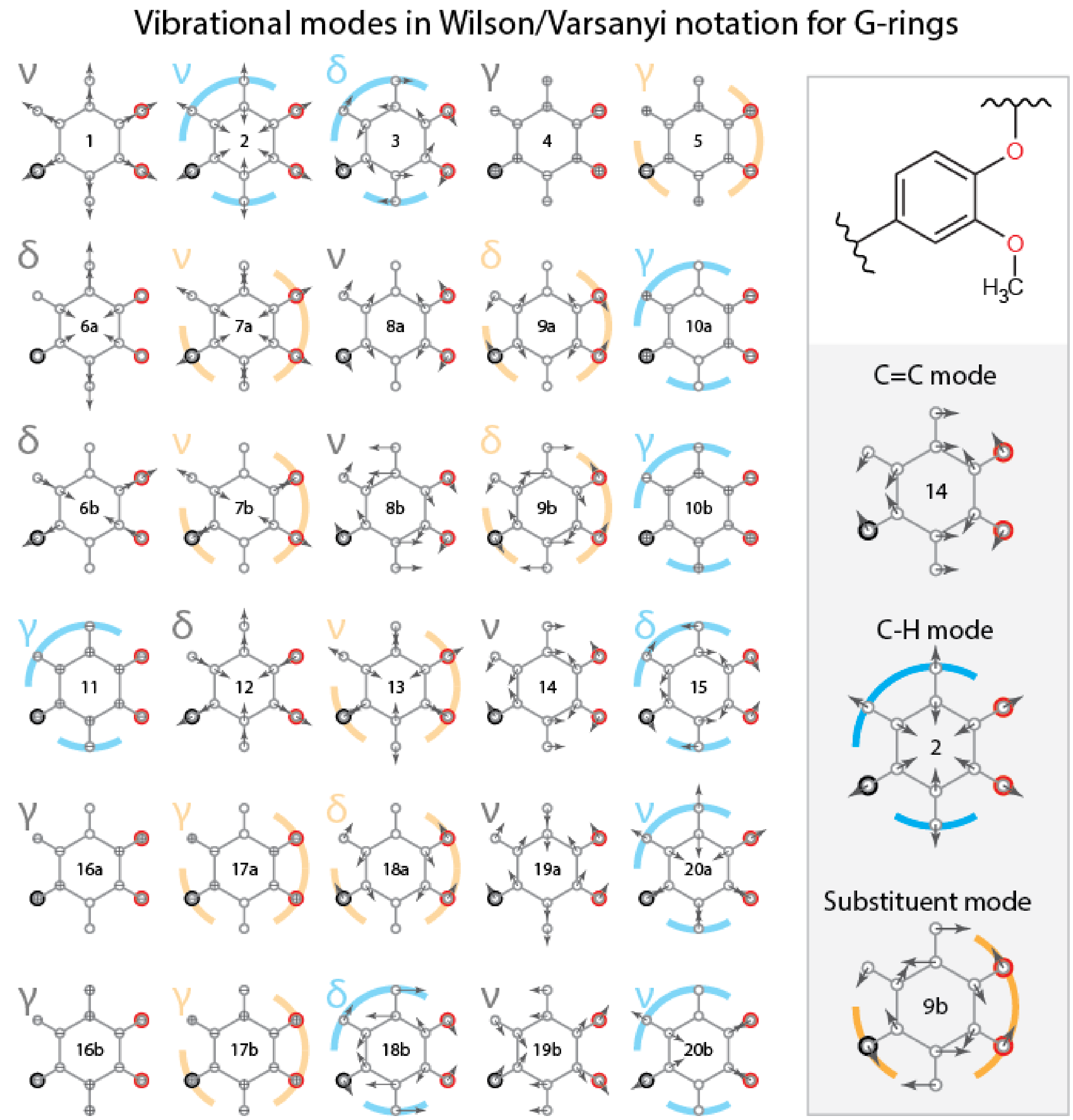

Figure 30 - Vibrational modes in Wilson[35]/Varsanyi[12] notation for G-rings. The 30 modes of benzene are divided into 12 ring carbon modes, 9 hydrogen modes and 9 substituent modes. Arrows depict atomic displacements in the plane of the paper and + and – indicate motion out of the paper plane. The magnitude and direction of displacement have only illustrative character. Calculated displacements of actual molecules can considerably deviate from those shown here, although the principal character of the mode can normally still be recognized.

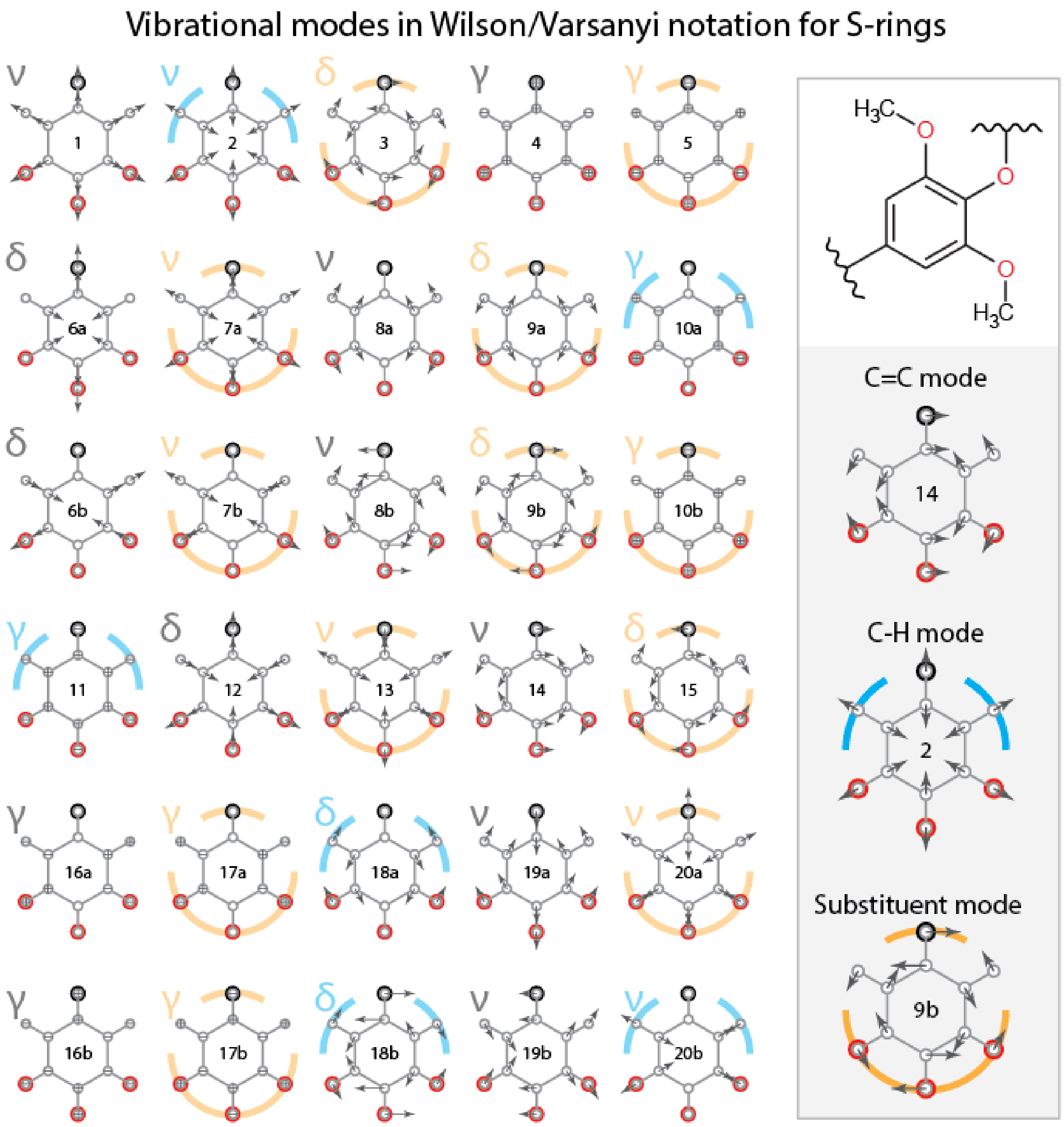

Figure 31 - Vibrational modes in Wilson[35]/Varsanyi[12] notation for S-rings. The 30 modes of benzene are divided into 12 ring carbon modes, 6 hydrogen modes and 12 substituent modes. Arrows depict atomic displacements in the plane of the paper and + and – indicate motion out of the paper plane. The magnitude and direction of displacement have only illustrative character. Calculated displacements of actual molecules can considerably deviate from those shown here, although the principal character of the mode can normally still be recognized. Note that, although shown for S-rings, this chart is valid for any asym-tetrasubstitution and can therefore be applied to the individual rings of the biphenyl unit as well.

Literature

[1] M. W. Schmidt, K. K. Baldridge, J. A. Boatz, S. T. Elbert, M. S. Gordon, J. H. Jensen, S. Koseki, N. Matsunaga, K. A. Nguyen, S. J. Su, T. L. Windus, M. Dupuis, J. A. Montgomery, *Comput. Chem.* **1993**, *14*, 16.
[2] M. S. Gordon, M. W. Schmidt in *Theory and Applications of Computational Chemistry: the first forty years*; Dykstra CE, Frenking G, Kim KS, Scuseria GE, Eds.; Elsevier: Amsterdam, **2005**, p 19.
[3] B. M. Bode, M. S. Gordon, *J. Mol. Graphics Mod.* **1998**, *16*, 6.
[4] G. W. T. M. J. Frisch, H. B. Schlegel, G. E. Scuseria, M. A. Robb, J. R. Cheeseman, G. Scalmani, V. Barone, G. A. Petersson, H. Nakatsuji, X. Li, M. Caricato, A. V. Marenich, J. Bloino, B. G. Janesko, R. Gomperts, B. Mennucci, H. P. Hratchian, , A. F. I. J. V. Ortiz, J. L. Sonnenberg, D. Williams-Young, F. Ding, F. Lipparini, F. Egidi, J. Goings, B. Peng, A. Petrone, T. Henderson, D. Ranasinghe, V. G. Zakrzewski, J. Gao, N. Rega, , W. L. G. Zheng, M. Hada, M. Ehara, K. Toyota, R. Fukuda, J. Hasegawa, M. Ishida, T. Nakajima, Y. Honda, O. Kitao, H. Nakai, T. Vreven, K. Throssell, J. A. Montgomery, Jr., J. E. Peralta, F. Ogliaro, M. J. Bearpark, J. J. Heyd, E. N. Brothers, K. N. Kudin, , T. A. K. V. N. Staroverov, R. Kobayashi, J. Normand, K. Raghavachari, A. P. Rendell, J. C. Burant, S. S. Iyengar, J. Tomasi, M. Cossi, J. M. Millam, M. Klene, C. Adamo, R. Cammi, , R. L. M. J. W. Ochterski, K. Morokuma, O. Farkas, J. B. Foresman, and D. J. Fox; Gaussian, Inc.: Wallingford CT, **2016**.
[5] Wavefunction Inc.: Irvine CA, **2016**.
[6] P. Bock, N. Gierlinger, *Journal of Raman Spectroscopy* **2019**, *50*, 778.
[7] A. Lahdetie, P. Nousiainen, J. Sipila, T. Tamminen, A. S. Jaaskelainen, *Holzforschung* **2013**, *67*, 531.
[8] N. B. Colthup, L. H. Daly, S. E. Wiberley, *Introduction to Infrared and Raman Spectroscopy*; 3rd Edition ed.; Academic Press Inc.: New York, **1990**, 547.
[9] J. Jia, H.-S. Wu, Z. Chen, Y. Mo, *European Journal of Organic Chemistry* **2013**, *2013*, 611.
[10] A. Almenningen, O. Bastiansen, L. Fernholt, S. Gundersen, E. Klosterjensen, B. N. Cyvin, S. J. Cyvin, S. Samdal, A. Skancke, *Journal of Molecular Structure* **1985**, *128*, 77.
[11] Dannibal.A, L. Lunazzi, A. C. Boicelli, D. Macciantelli, *Journal of the Chemical Society-Perkin Transactions 2* **1973**, 1396.
[12] G. Varsanyi, *Vibrational Spectra of Benzene Derivatives*; 1st Edition ed.; Academic Press: New York, **1969**.
[13] B. Z. Chowdhry, J. P. Ryall, T. J. Dines, A. P. Mendham, *J Phys Chem A* **2015**, *119*, 11280.
[14] M. Felhofer, B. Prats-Mateu, P. Bock, N. Gierlinger, *Tree Physiol.* **2018**, *38*, 1.
[15] E. L. Sibert, D. P. Tabor, N. M. Kidwell, J. C. Dean, T. S. Zwier, *Journal of Physical Chemistry A* **2014**, *118*, 11272.
[16] L. J. Bellamy, D. W. Mayo, *Journal of Physical Chemistry* **1976**, *80*, 1217.
[17] D. W. Mayo, F. A. Miller, R. W. Hannah, *Course Notes on the Interpretation of Infrared and Raman Spectra*; John Wiley & Sons, Inc: Hoboken, New Jersey, **2003**.
[18] D. C. Mckean, *Chemical Society Reviews* **1978**, *7*, 399.
[19] I. A. Degen, *Appl Spectrosc* **1986**, *22*, 3.
[20] R. J. Gillespie, R. S. Nyholm, *Quarterly Reviews* **1957**, *11*, 339.
[21] R. J. Gillespie, *J. Chem. Educ.* **1963**, *40*, 7.
[22] R. J. Gillespie, *Journal of Chemical Education* **1974**, *51*, 367.
[23] A. D. Clauss, S. F. Nelsen, M. Ayoub, J. W. Moore, C. R. Landis, F. Weinhold, *Chemistry Education Research and Practice* **2014**, *15*, 417.
[24] P. C. Hiberty, D. Danovich, S. Shaik, *Chemistry Education Research and Practice* **2015**, *16*, 689.

[25] J. T. Braunholtz, E. A. V. Ebsworth, F. G. Mann, N. Sheppard, *Journal of the Chemical Society* **1958**, 2780.
[26] H. P. Hamlow, S. Okuda, *Tetrahedron Letters* **1964**, 2553.
[27] M. Rumi, G. Zerbi, *Journal of Molecular Structure* **1999**, *509*, 11.
[28] S. A. Katsyuba, R. Schmutzler, U. Hohm, C. Kunze, *Journal of Molecular Structure* **2002**, *610*, 113.
[29] L. J. H. Hoffmann, S. Marquardt, A. S. Gemechu, H. Baumgartel, *Physical Chemistry Chemical Physics* **2006**, *8*, 2360.
[30] P. Karhunen, P. Rummakko, A. Pajunen, G. Brunow, *Journal of the Chemical Society-Perkin Transactions 1* **1996**, 2303.
[31] P. P. Shorygin, *Russian Chem. Reviews* **1971**, *40*, 367.
[32] E. D. Schmid, B. Brosa, *Berichte Der Bunsen-Gesellschaft Fur Physikalische Chemie* **1971**, *75*, 1334.
[33] E. D. Schmid, R. D. Topsom, *Journal of the American Chemical Society* **1981**, *103*, 1628.
[34] E. D. Schmid, B. Brosa, *Journal of Chemical Physics* **1973**, *58*, 3871.
[35] E. B. Wilson, *Phys. Rev.* **1934**, *45*, 706.
